# Supplementary material for: CRISPR-Cas9 cytidine and adenosine base editing of splice-sites mediates highly-efficient disruption of proteins in primary and immortalized cells
Source: Nat Commun. 2021 Apr 23;12:2437. doi: 10.1038/s41467-021-22009-2 (PMC8065034; doi:10.1038/s41467-021-22009-2)
Supplement: Supplementary file 1 — Supplementary Information [file 41467_2021_22009_MOESM1_ESM.pdf]

CRISPR-Cas9 cytidine and adenosine base editing of splice-sites mediates highly-efficient disruption of proteins in primary and immortalized cells

Supplementary Figures
Updated March 24th, 2021

AUTHORS

Mitchell G. Kluesner[1,2,3,4*], Walker S. Lahr[1,2,3,4,*] Cara-lin Lonetree[1,2,3,4], Branden A. Smeester[1,2,3,4], Xiaohong Qiu[1,2,3,4], Nicholas J. Slipek[1,2,3,4], Patricia N. Claudio Vázquez[1,2,3,4,5], Samuel P. Pitzen[2,5], Emily J. Pomeroy[1,2,3,4], Madison J. Vignes[6], Samantha C. Lee[5,6], Samuel P. Bingea[1,2,3,4], Aneesha A. Andrew[5,6], Beau R. Webber[1,2,3,4,†], Branden S. Moriarity[1,2,3,4,†]

*These authors contributed equally
†These authors contributed equally

AFFILIATIONS
[1]Department of Pediatrics, University of Minnesota, Minneapolis, MN, USA
[2]Masonic Cancer Center, University of Minnesota, Minneapolis, MN, USA
[3]Center for Genome Engineering, University of Minnesota, Minneapolis, MN, USA
[4]Stem Cell Institute, University of Minnesota, Minneapolis, MN, USA
[5]Department of Genetics, Cell Biology, and Development, University of Minnesota, Minneapolis, MN, USA
[6]College of Biological Sciences, University of Minnesota, Minneapolis, MN, USA

Supplementary Figure S1

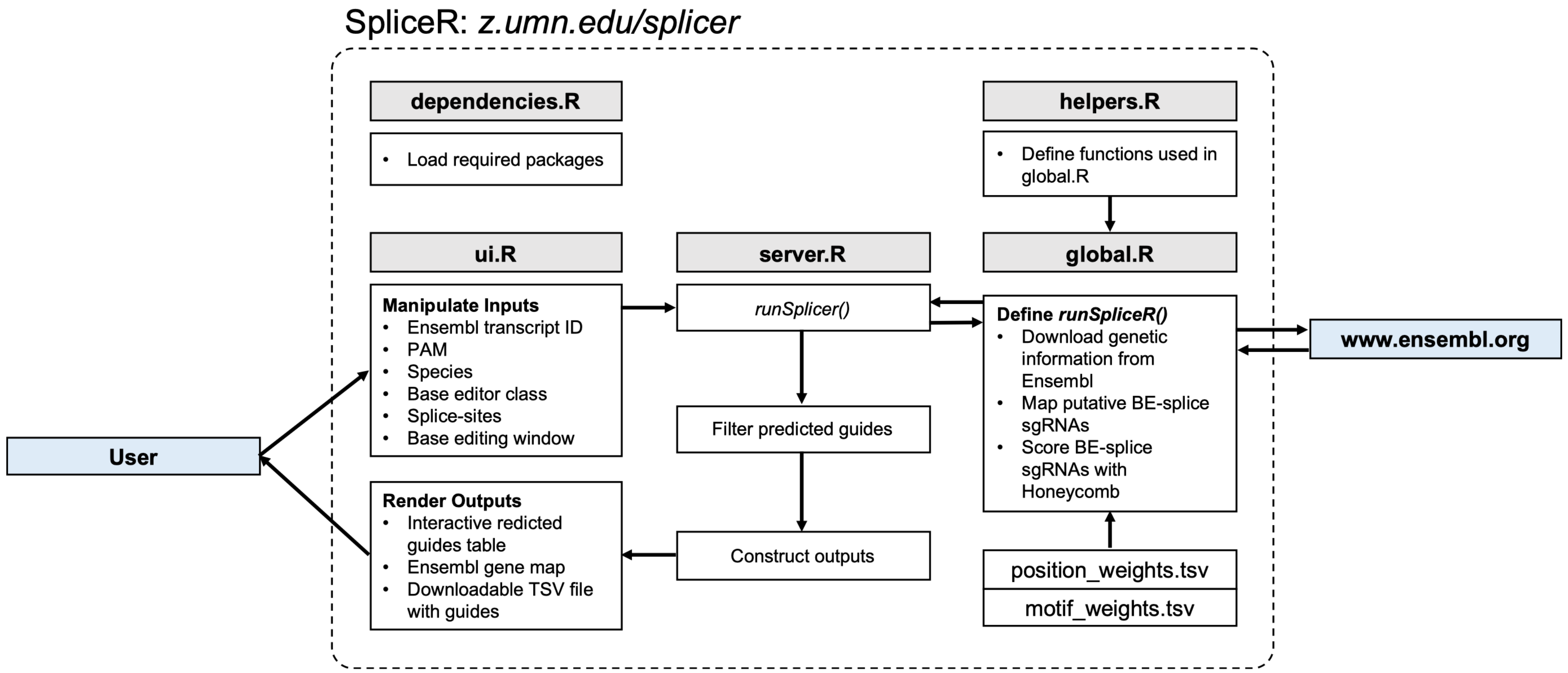

Supplementary Figure S1. Diagram of SpliceR v2.0.0. User provides an Ensembl transcript ID, a preferred PAM, species being targeted, base editor class, desired splice-sites to target, and a base editing window. SpliceR communicates directly with Ensembl to pull genetic information and map guides. sgRNAs are then scored by context motif and position of target base in the protopsacer. Users can download predicted sgRNAs.

Supplementary Figure S2

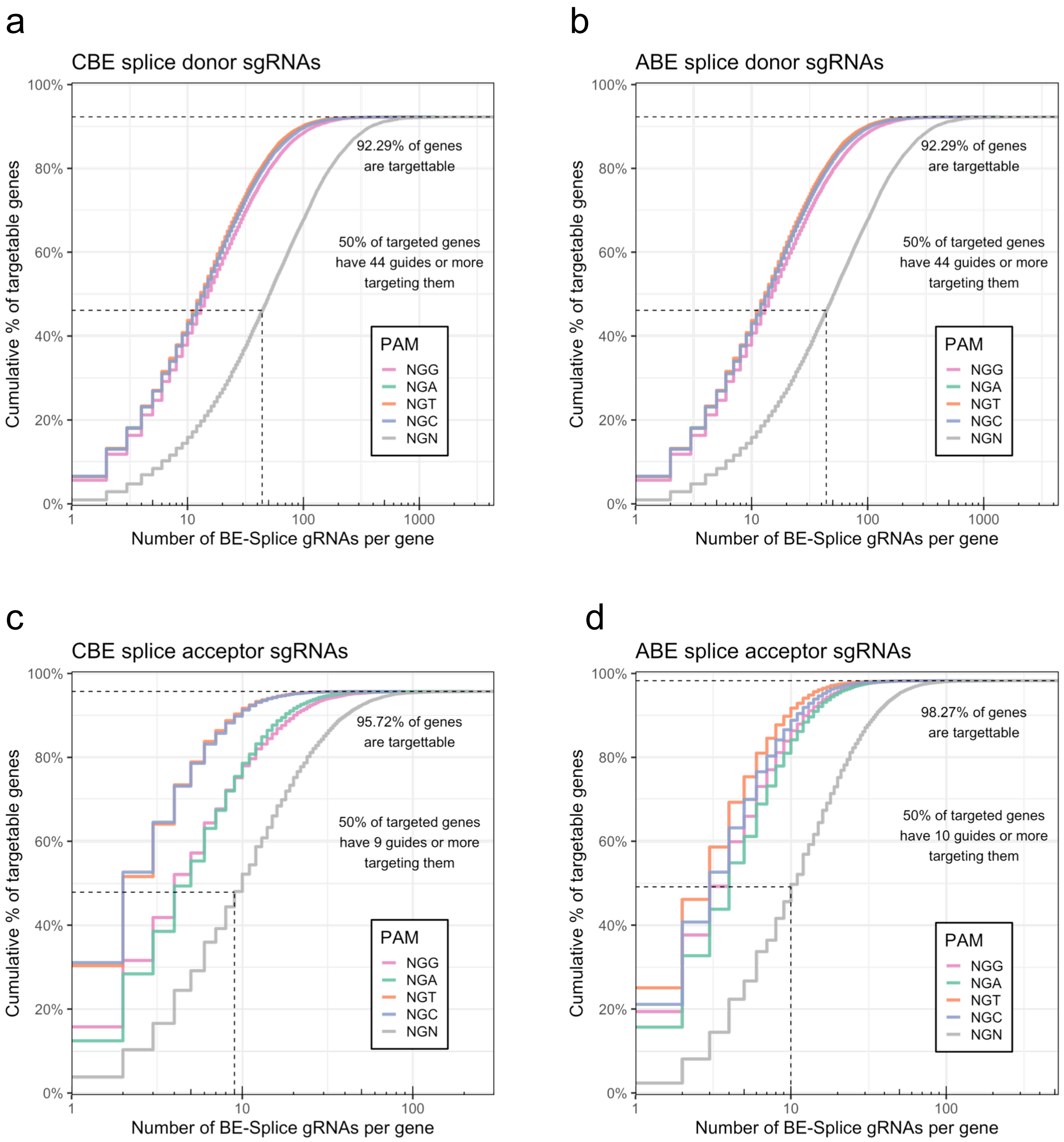

Supplementary Figure S2. Distribution of BE-splice sgRNA density across all genes by BE-splice approach. (a) CBE splice donors, (b) ABE splice donors, (c) CBE splice acceptors, (d) ABE splice acceptors. Note that CBE and ABE splice donors utilize the same sgRNAs, hence the same guide density.

Supplementary Figure S3

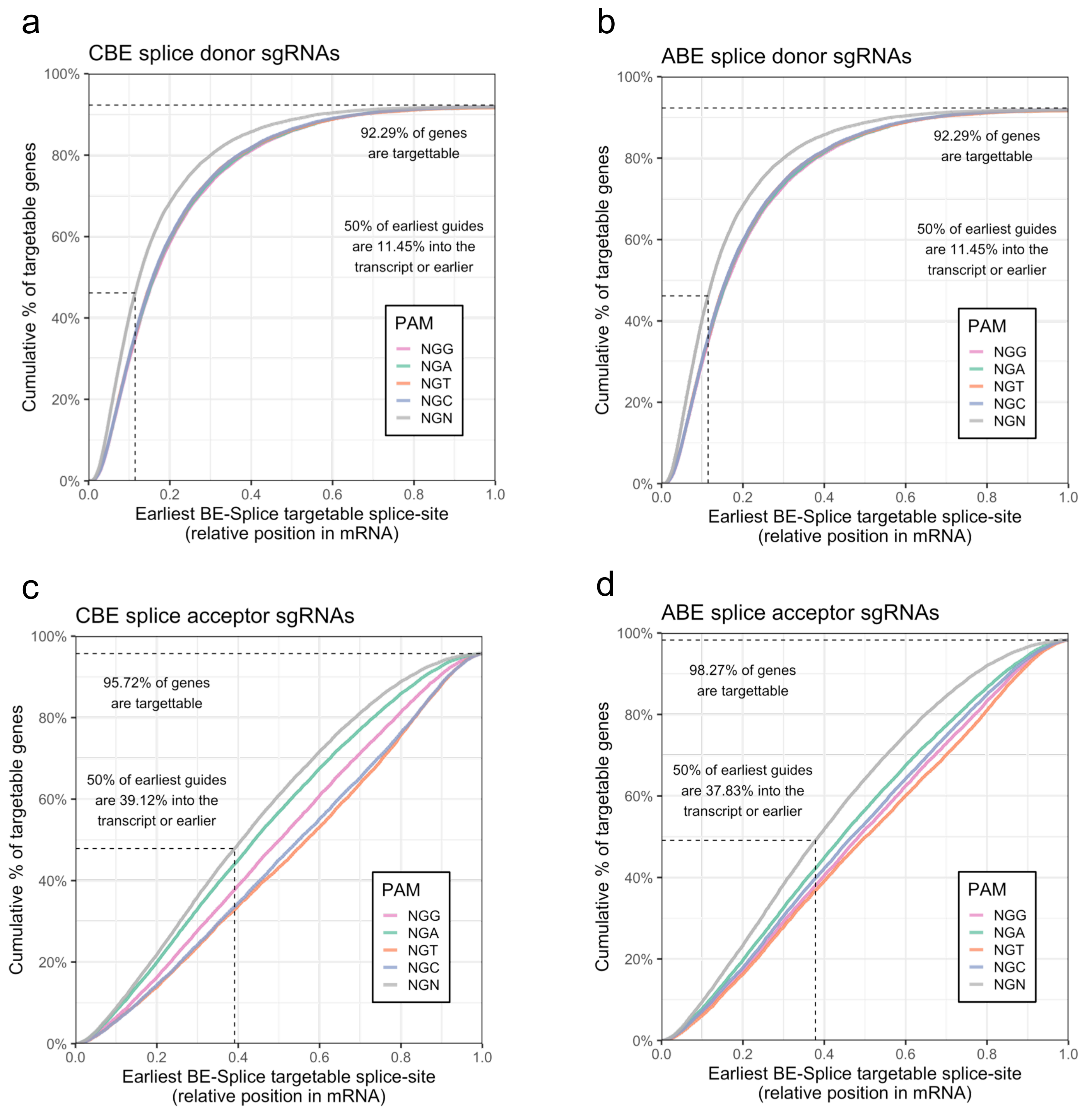

Supplementary Figure S3. Distribution of the position of the first sgRNA across all genes by BE-splice approach. (a) CBE splice donors, (b) ABE splice donors, (c) CBE splice acceptors, (d) ABE splice acceptors. Note that CBE and ABE splice donors utilize the same sgRNAs, hence the same guide density.

Supplementary Figure S4

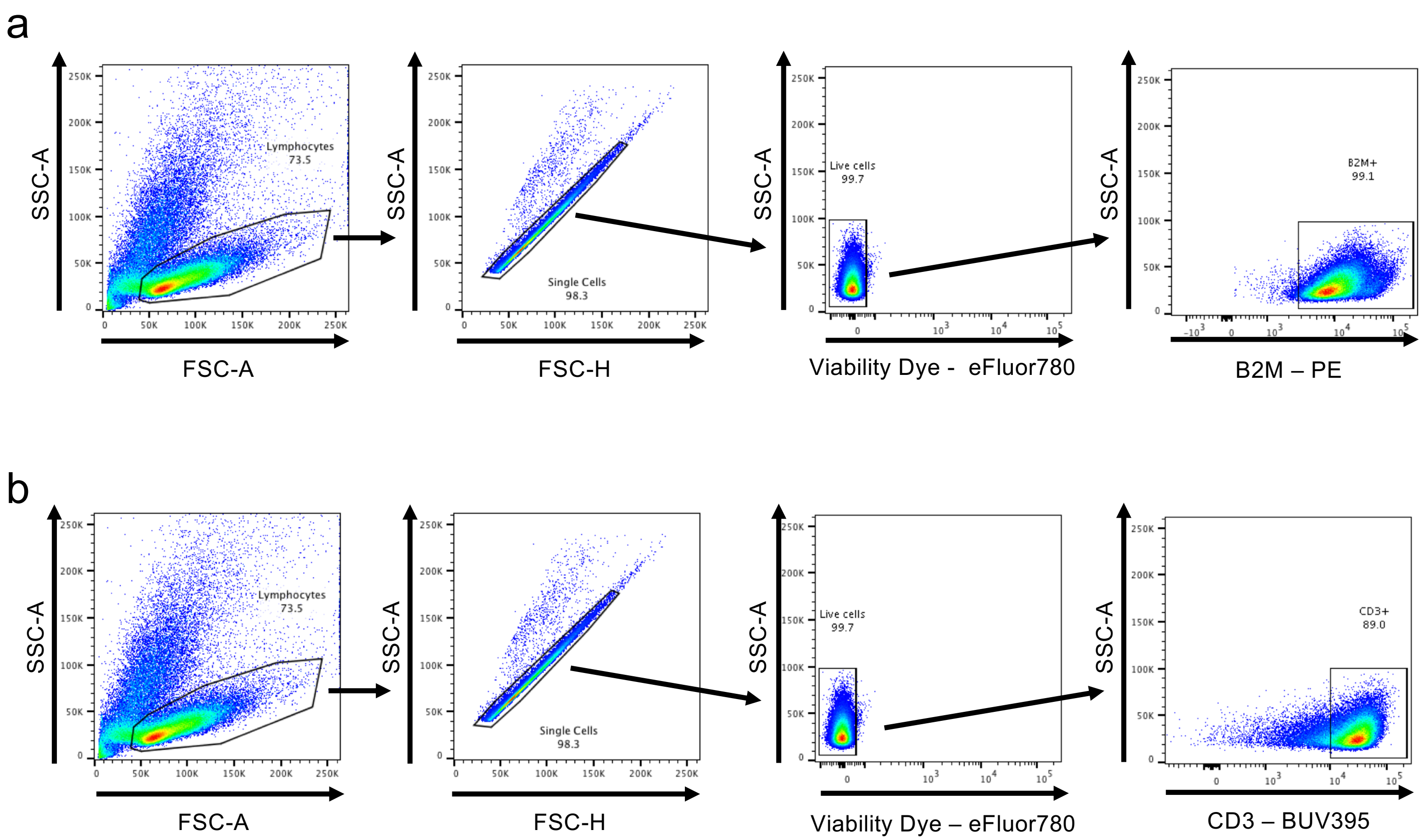

Supplementary Figure S4. Representative gating strategies for flow cytometry. (a) Gating tree for B2M$^+$ cells. (b) Gating tree for CD3$^+$ cells. Gating strategy applied to data in Figure 2, 3, and 5.

Supplementary Figure S5

a

Dinucleotide	Average Editing	Std. Error	t value	Pr(>|t|)
All	17.4%	1.42%	12.20	0.00e+00
TC	28.7%	3.07%	9.33	0.00e+00
CC	21.1%	2.68%	7.88	0.00e+00
AC	13.8%	2.91%	4.73	2.70e-06
GC	6.9%	3.05%	2.25	2.48e-02

b

Dinucleotide	Average Editing	Std. Error	t value	Pr(>|t|)
All	12.6%	1.33%	9.45	0.000000
TA	24.5%	2.93%	8.37	0.000000
CA	13.5%	2.73%	4.93	0.000001
AA	10.2%	2.70%	3.78	0.000170
GA	6.3%	2.47%	2.55	0.010800

c

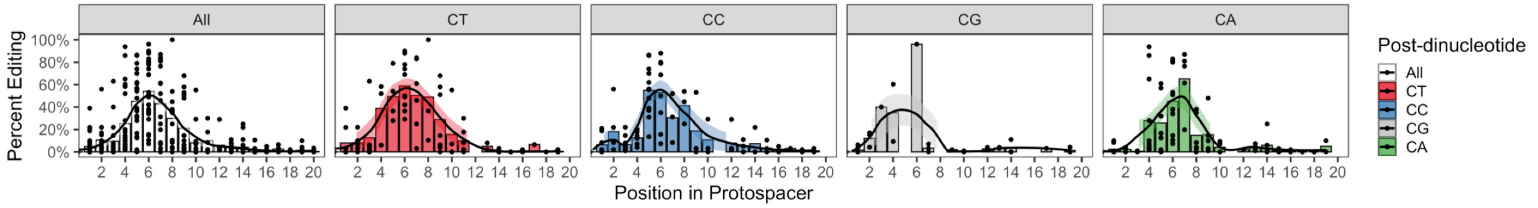

d

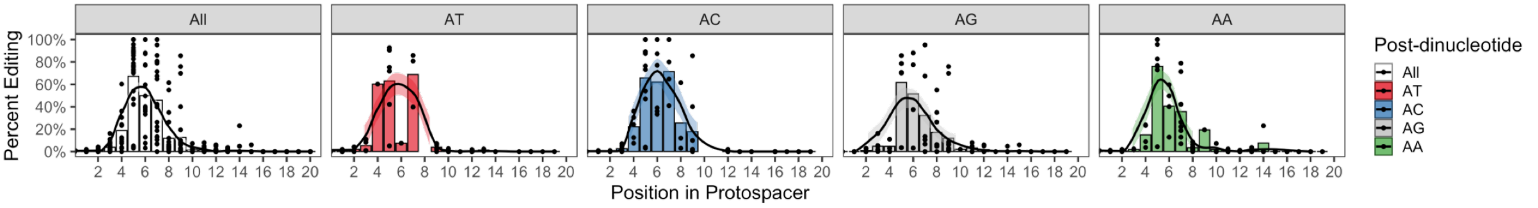

e

PostDinucleotide	Average Editing	Std. Error	t value	Pr(>|t|)
All	17.4%	1.45%	12.00	0.0000
CT	21.5%	2.71%	7.92	0.0000
CC	19.6%	2.71%	7.22	0.0000
CA	8.5%	4.38%	1.93	0.0534
CG	17.0%	2.71%	6.24	0.0000

f

PostDinucleotide	Average Editing	Std. Error	t value	Pr(>|t|)
All	12.6%	1.34%	9.35	0.00e+00
AT	10.7%	3.24%	3.29	1.04e-03
AC	20.1%	2.84%	7.10	0.00e+00
AA	9.0%	2.35%	3.82	1.46e-04
AG	13.5%	2.73%	4.94	9.00e-07

Supplementary Figure S5. Dinucleotide context dependencies of rAPOBEC1-BE4 and $TadA^{WT}$-$TadA^{Evo}$-ABE7.10. (a) Summary of linear model of rAPOBEC1-BE4 editing efficiency as a function of pre-dinucleotide context. (b) Summary of linear model of $TadA^{WT}$-$TadA^{Evo}$-ABE7.10 editing efficiency as a function of pre-dinucleotide context. (c) Distribution of APOBEC1-BE4 editing efficiency across the protospacer by post-dinucleotide context. (d) Distribution of $TadA^{WT}$-$TadA^{Evo}$-ABE7.10 editing efficiency across the protospacer by post-dinucleotide context. Note that distributions are not as smooth as pre-dinucleotide context (Fig. 4). (e) Summary of linear model of rAPOBEC1-BE4 editing efficiency as a function of post-dinucleotide context. (f) Summary of linear model of $TadA^{WT}$-$TadA^{Evo}$-ABE7.10 editing efficiency as a function of post-dinucleotide context.

Supplementary Figure S6

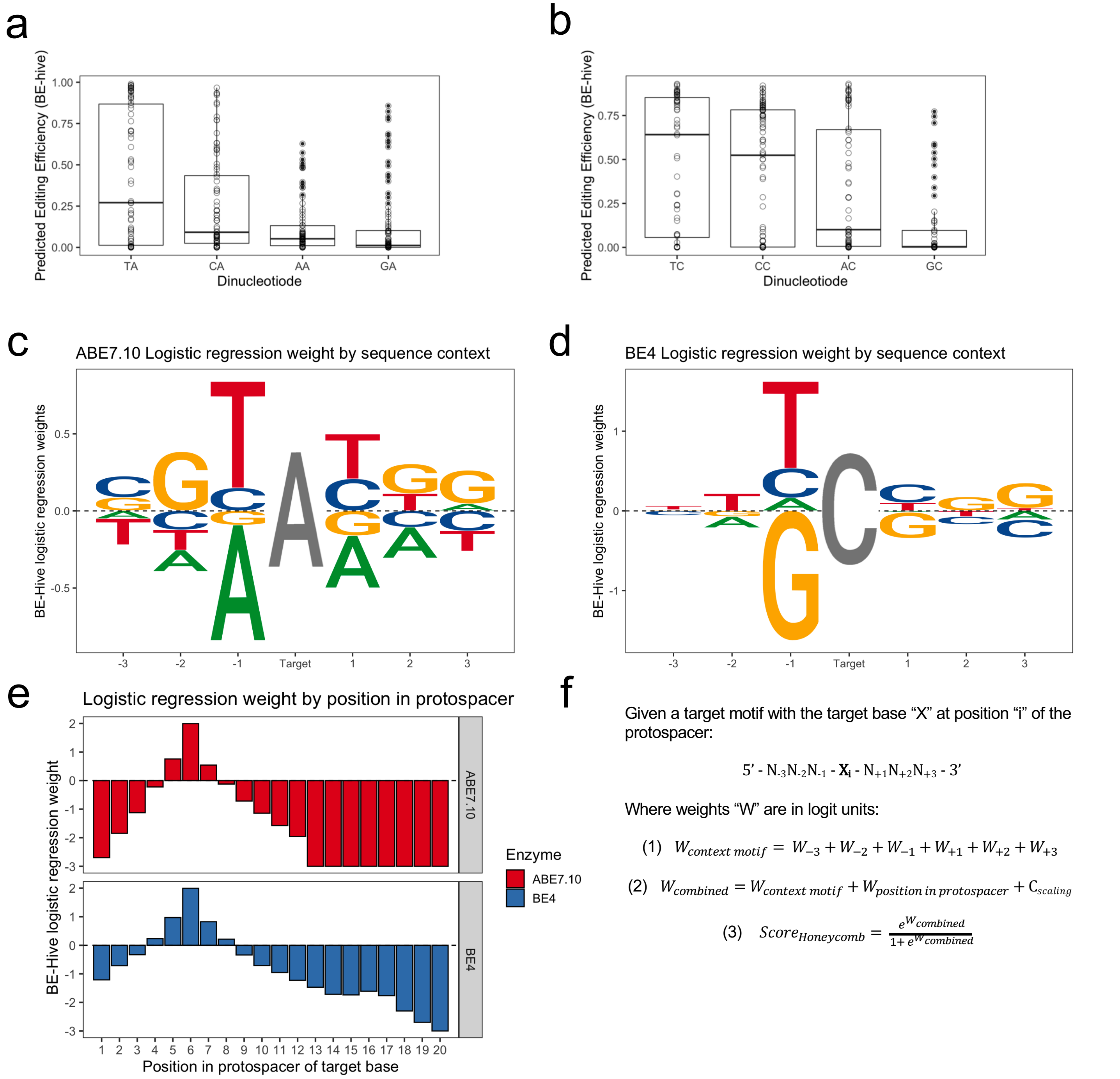

Supplementary Figure S6. Comparison of context preferences from meta-analysis to BE-hive and the basis of the Honeycomb scoring algorithm. (a-b) BE-Hive predicted editing efficiency for base edits in meta-analysis grouped by preceding dinucleotide context. Predicted results observe identical trends to empirical results from meta-analysis (Figure 4) for context specificities of ABE7.10 (left) and BE4 (right). Boxplot center lines represent the median, box limits represent the upper and lower quartiles, and whiskers define the 1.5x interquartile range. N = 6 papers, 102 guides, 447 edits in total. (c-d) logistic regression context motif weights of ABE7.10 and BE4 first demonstrated in Arbab & Shen et al.[40]. The height of the logos signify the weight magnitude of having a base identity at a particular position relative to the target base. The direction of the logo signifies whether having that base at that position increases or decreases the odds of editing. Target base is centered in grey. Preceding base context preferences recapitulate results from Fig. 4 a-b. (e) Logistic regression weights for ABE7.10 and BE4 by position of target base in the protospacer. ABE7.10 exhibits a narrowed window relative to BE4, recapitulating results from Fig. 4 a-c. (f) Calculations of Honeycomb score. Logistic weights are summed and transformed into a probability to generate a score. A scaling constant $C_{scaling}$ is added to equation (2) to allow for readily interpretable values. Addition of a scaling constant does not alter the rank of sgRNAs.

Supplementary Figure S7

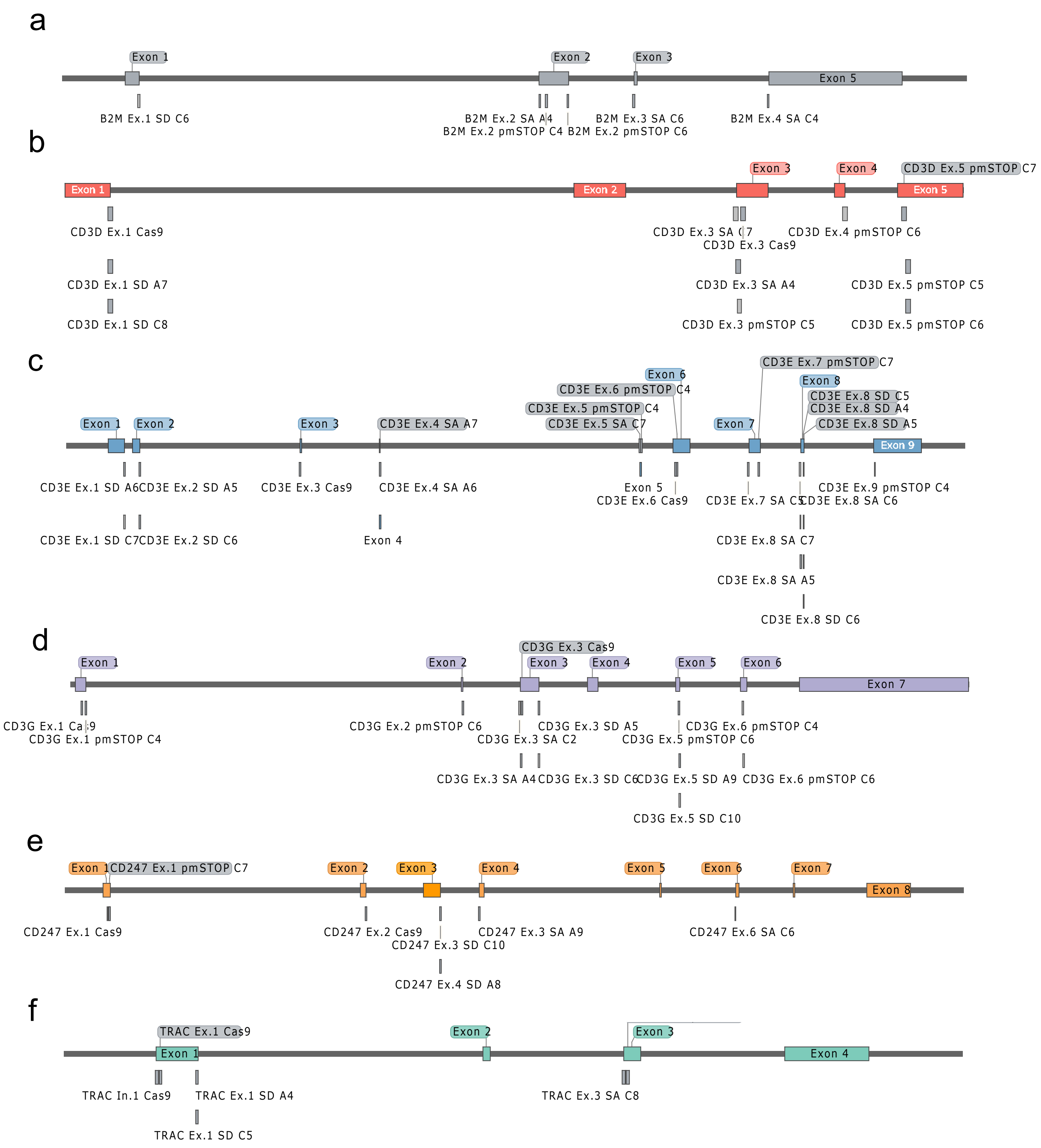

Supplementary Figure S7. Mapping of sgRNAs used in this work to the genomic loci of (a) B2M, (b) CD3D, (c) CD3E, (d) CD3G, (e) CD247, (f) TRAC. TRBC1 and TRBC2 were omitted from the BE-splice screen due to the inability to design single BE-splice sgRNAs to target both paralogs simultaneously.

Supplementary Figure S8

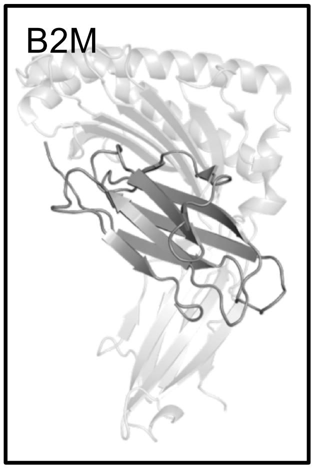

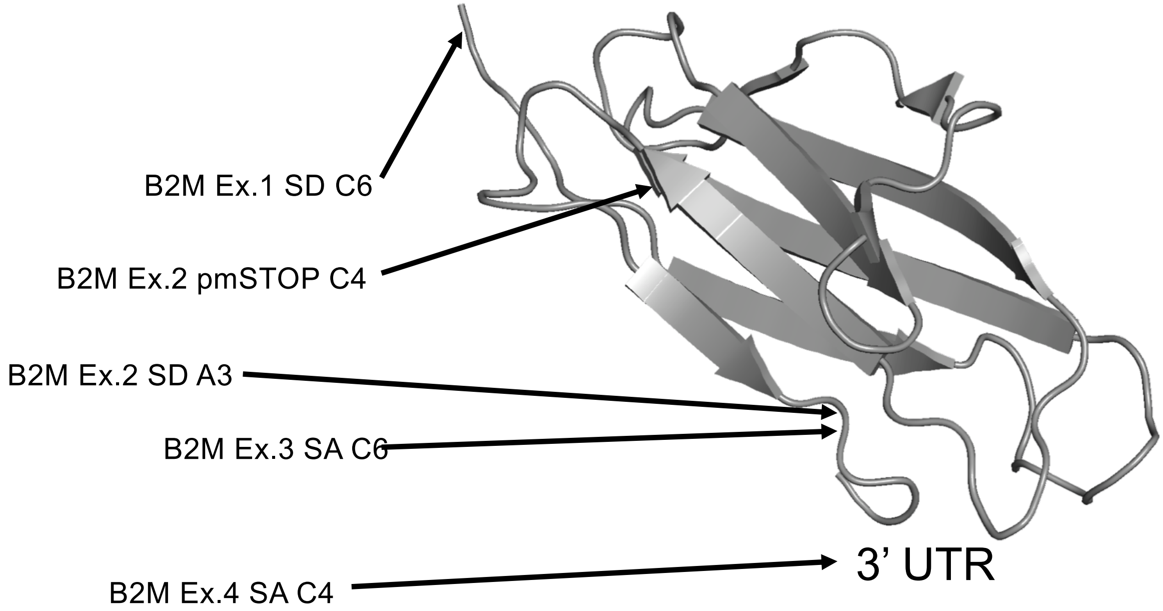

Supplementary Figure S8. Mapping of sgRNAs to B2M (β2M) protein structure (PDB 1OGA).

Supplementary Figure S9

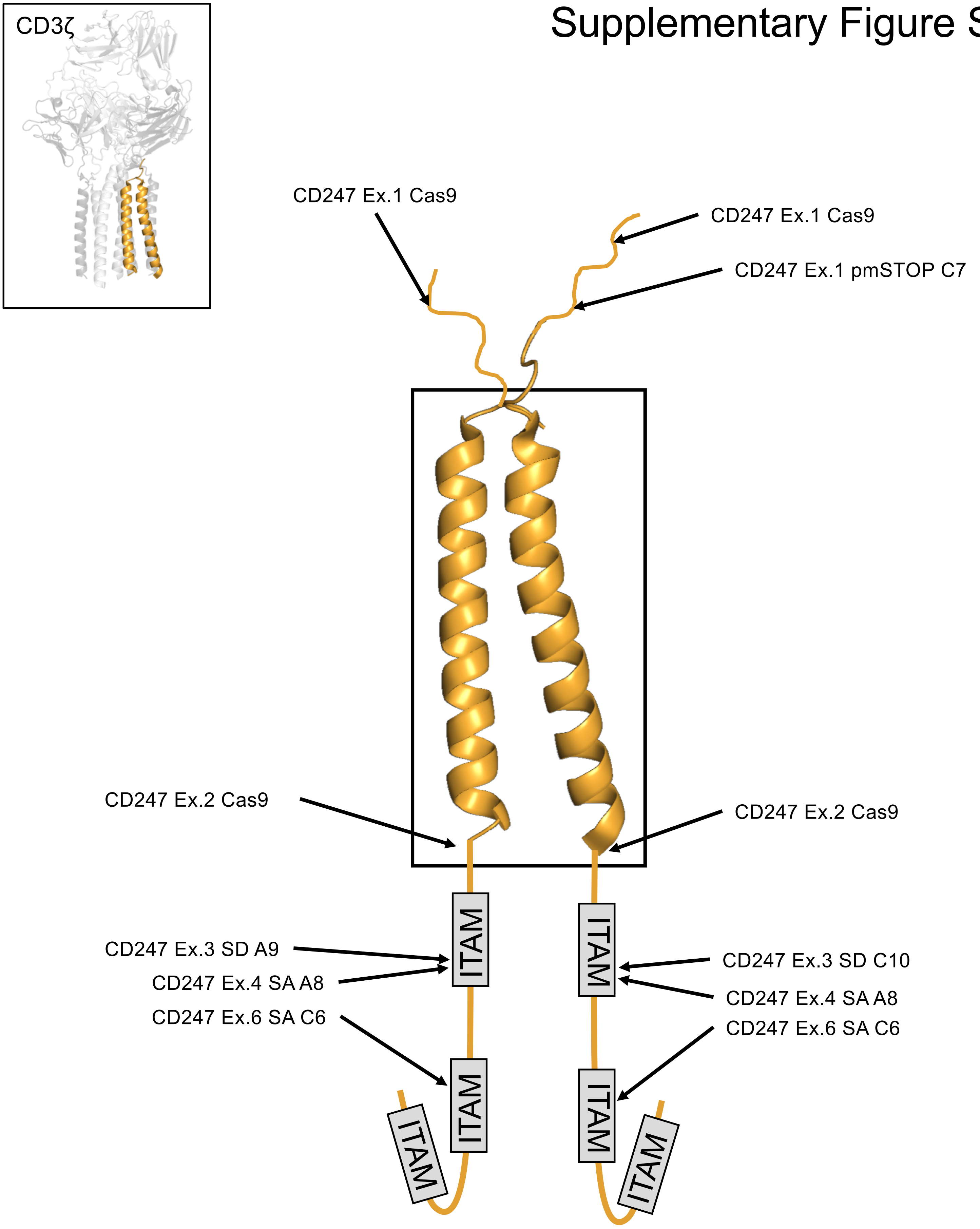

Supplementary Figure S9. Mapping of sgRNAs to CD247 (CD3ζ) protein structure (PDB 6JXR).

Supplementary Figure S10

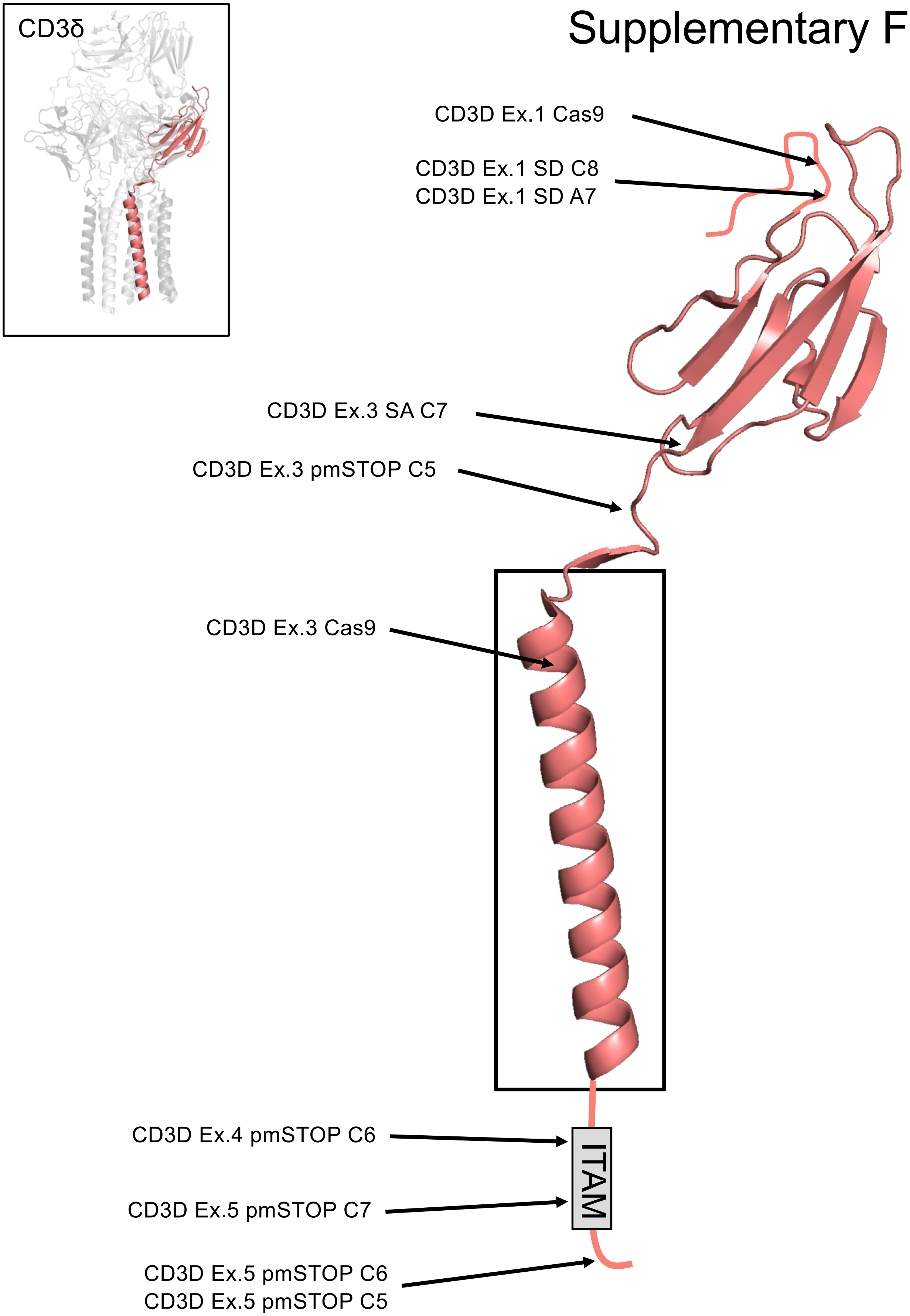

Supplementary Figure S10. Mapping of sgRNAs to CD3D protein structure (PDB 6JXR).

Supplementary Figure S11

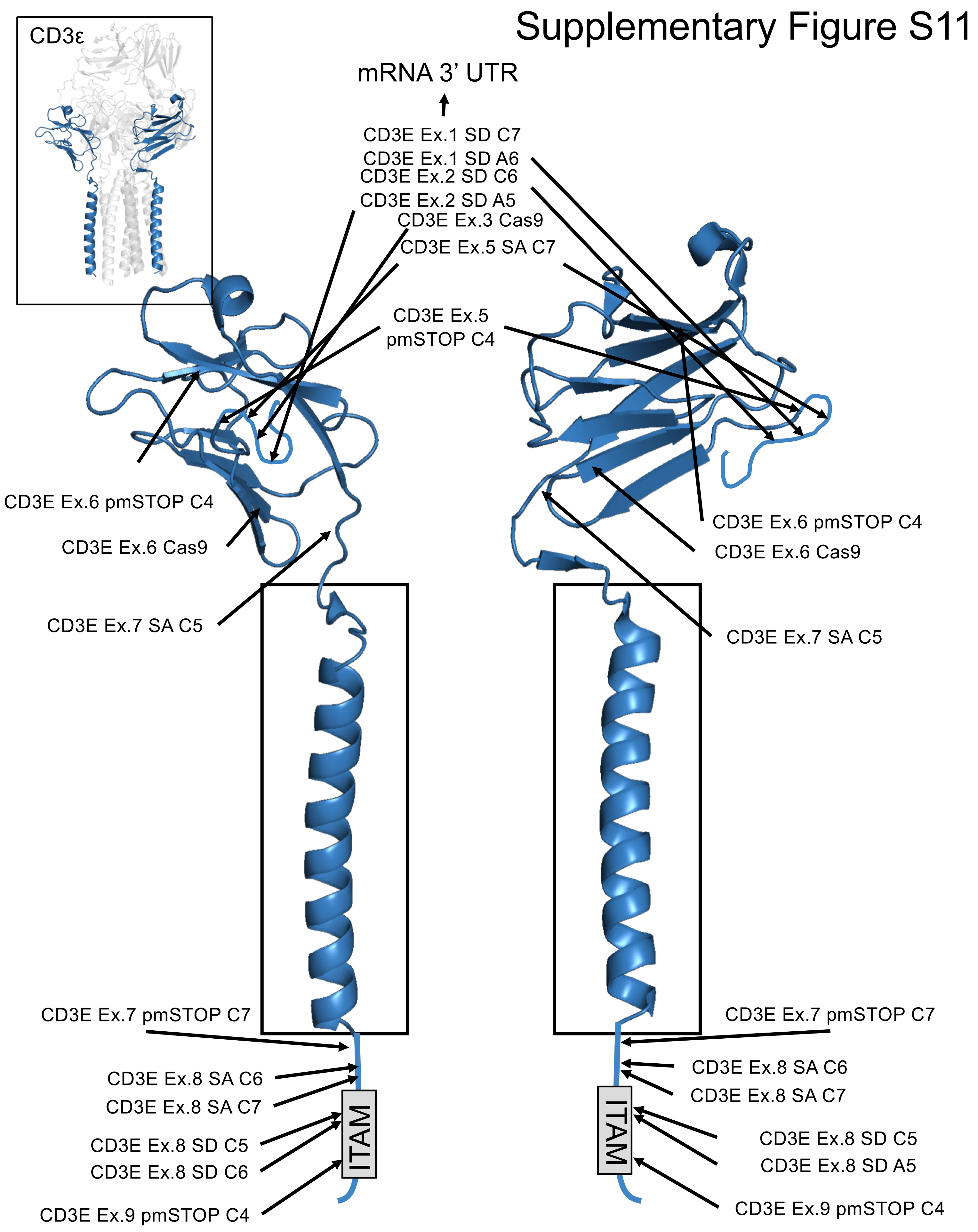

Supplementary Figure S11. Mapping of sgRNAs to CD3E protein structure (PDB 6JXR).

Supplementary Figure S12

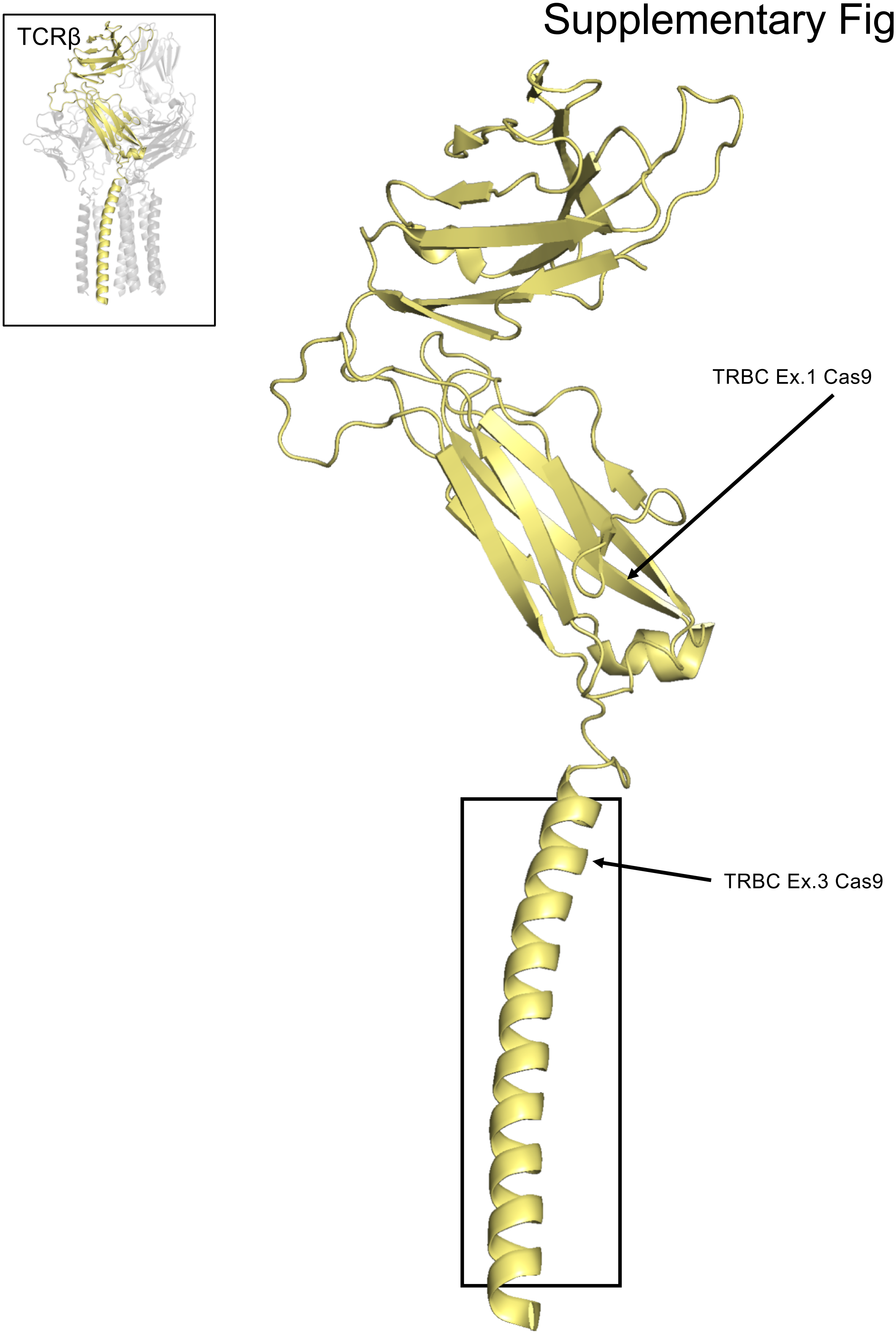

Supplementary Figure S12. Mapping of sgRNAs to TRBC (TCRβ) protein structure (PDB 6JXR).

Supplementary Figure S13

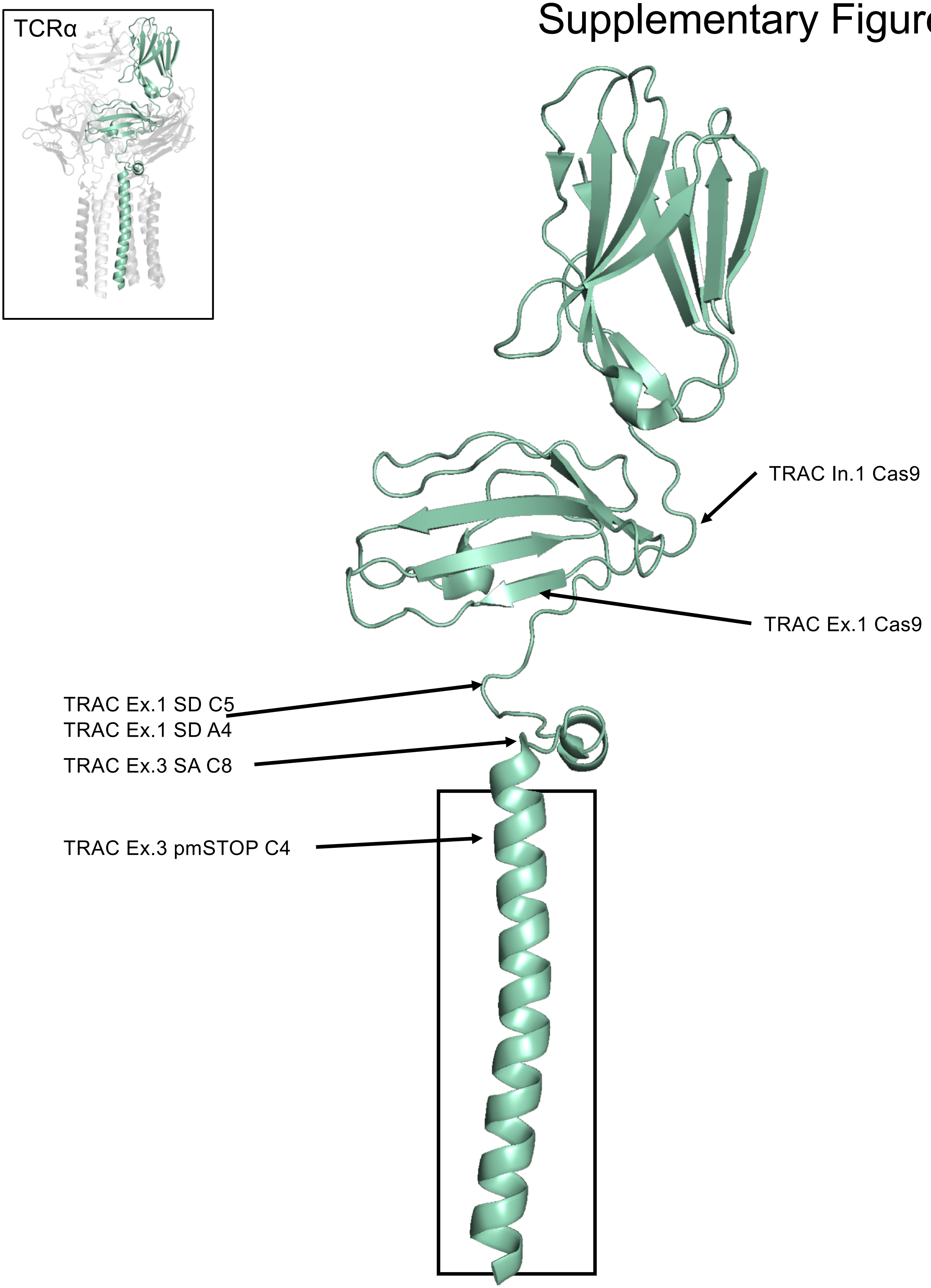

Supplementary Figure S13. Mapping of sgRNAs to TRAC (TCRα) protein structure (PDB 6JXR).

Supplementary Figure S14

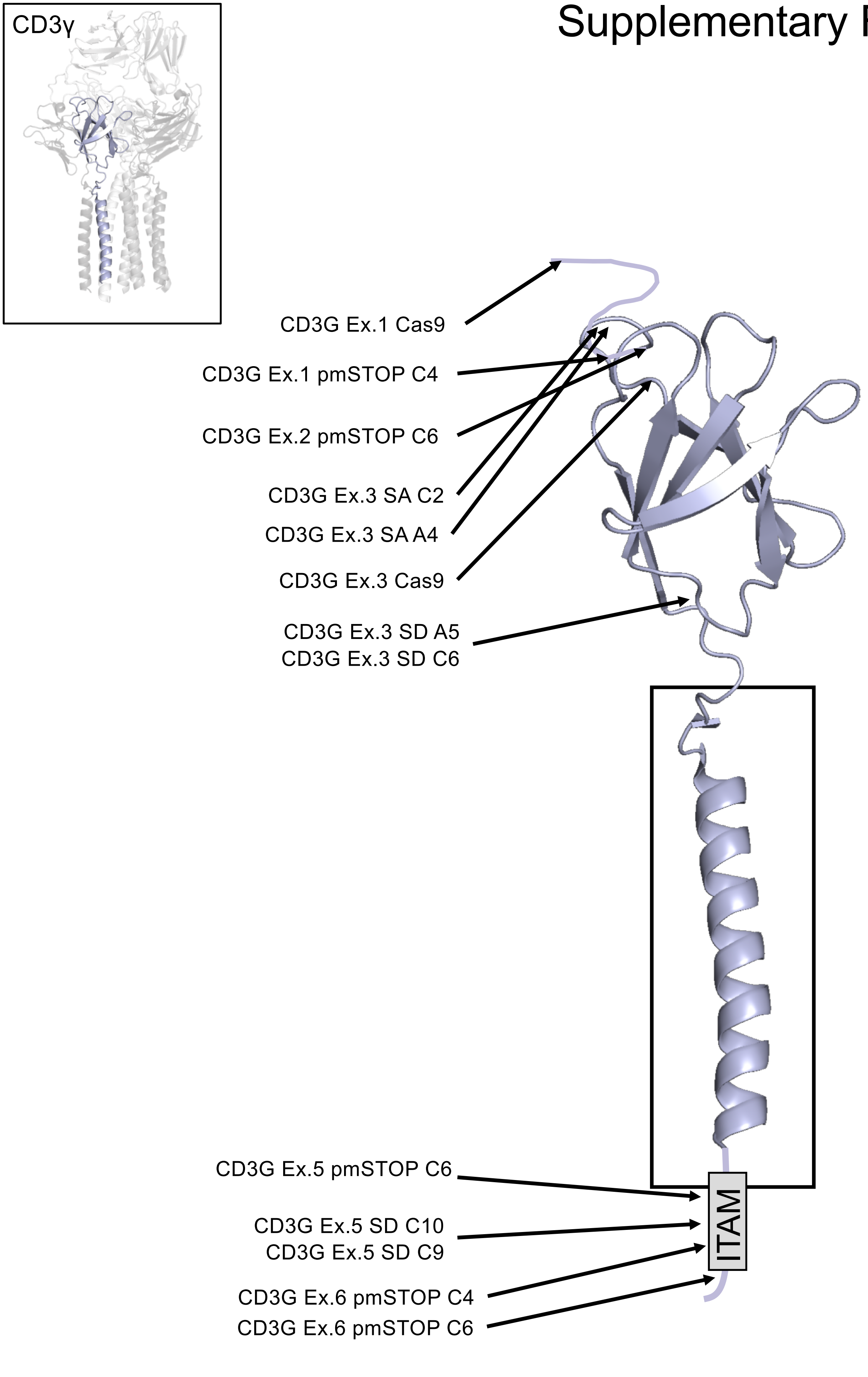

Supplementary Figure S14. Mapping of sgRNAs to CD3G protein structure (PDB 6JXR).

Supplementary Figure S15

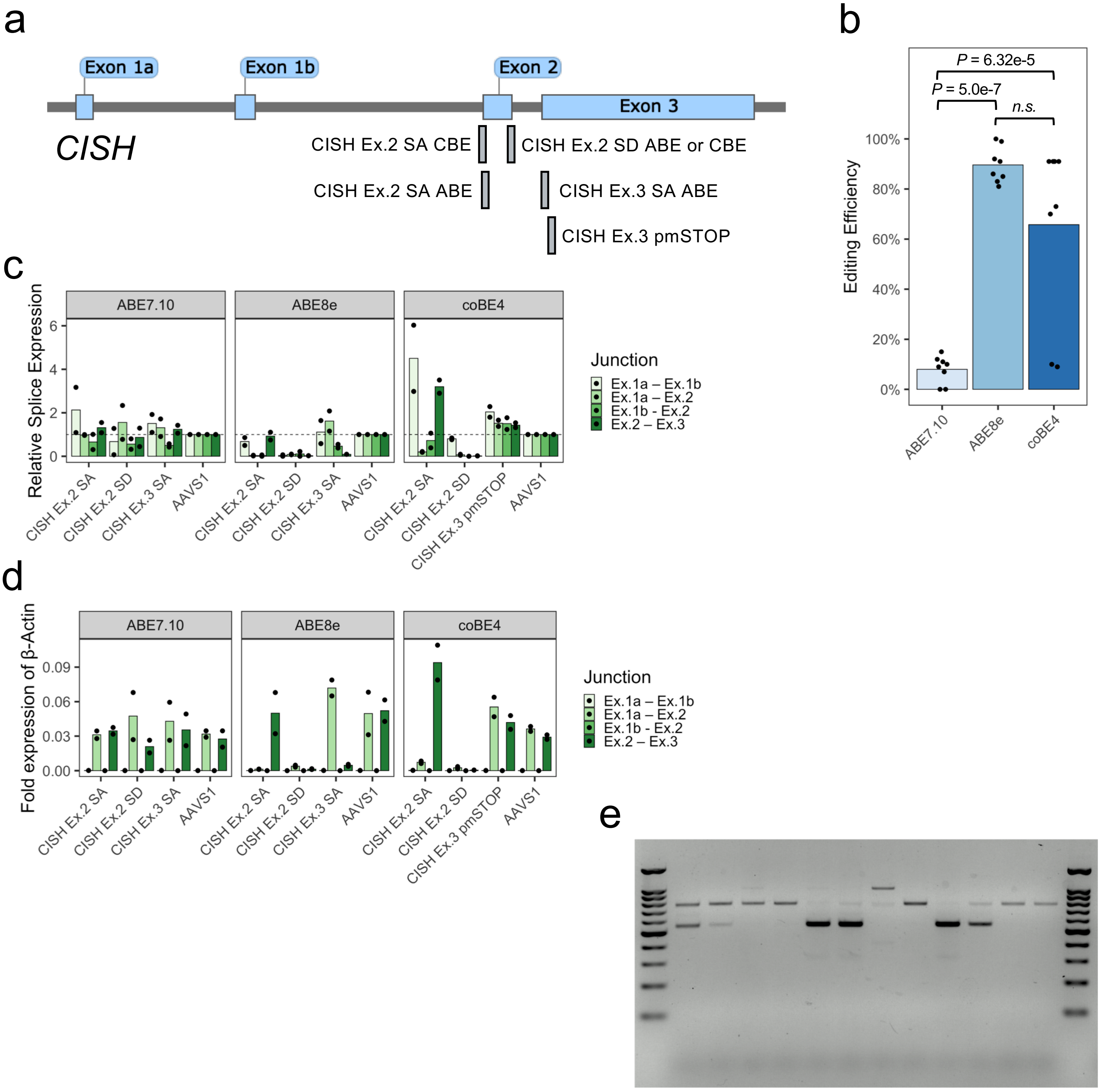

Supplementary Figure S15. Disrupting the immunoinhibitory protein CISH with BE-splice guides in K562 cell line. (a) Map of *CISH* locus with sgRNAs targeting known functional domains. (b) Comparison of editing efficiencies across ABE7.10, ABE8e, and BE4. N = 2 biological replicates. P-values represent results from one-way ANOVA followed by Tukey HSD test. (c) Relative splice site expression of all exon spanning Taqman assays across different treatments, N = 3 technical replicates per 2 biological replicates. (d) Expression of splice site junctions shown as the fold expression of β-Actin. Data demonstrates that the 1a-1b and 1b-2 junctions are nearly undetectable in K562, suggesting the presence of a single major CISH isoform. (e) Uncropped image of gel presented in figure 6e.

Supplementary Figure S16

a

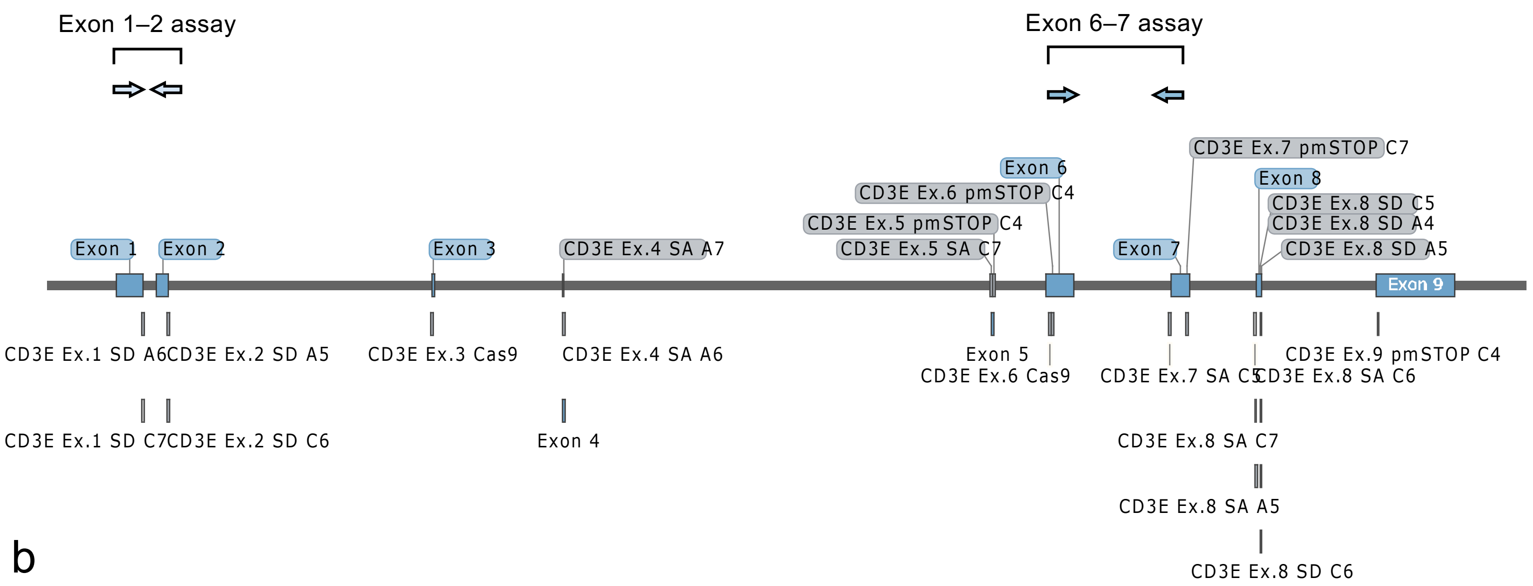

b

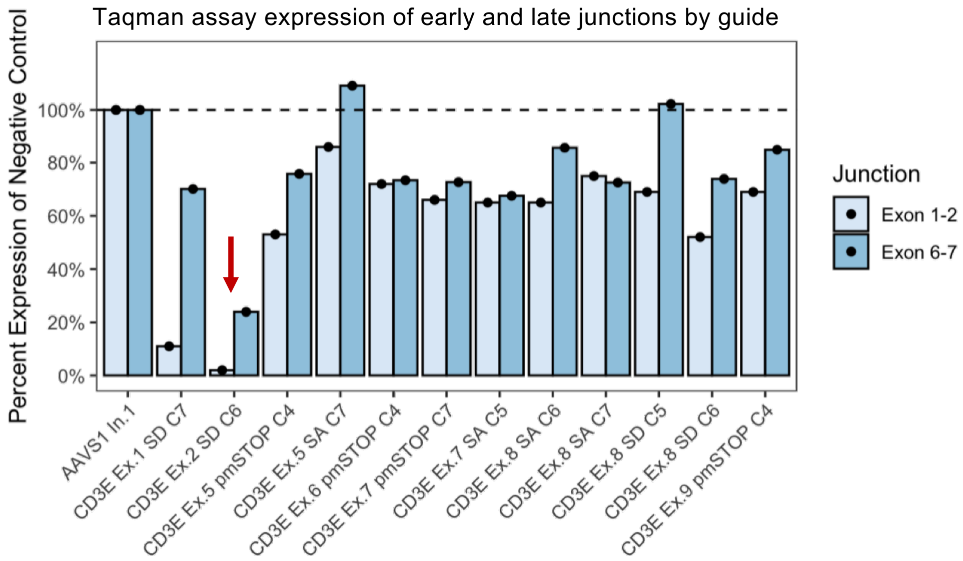

Supplementary Figure S16. Taqman assays for CD3E following treatment with various sgRNAs. (a) Map of CD3E locus and Taqman assays. (b) 2^-ΔΔCt Expression of Taqman assays normalized to AAVS1 control, expressed as a fold change. Arrow indicates sample with greatest decrease in Exon 1-2 and Exon 6-7 junction. (c) Corresponding remaining WT allele and protein expression of samples. Data suggests that CD3E Ex.2 SD targeting induces NMD of the entire transcript.

c

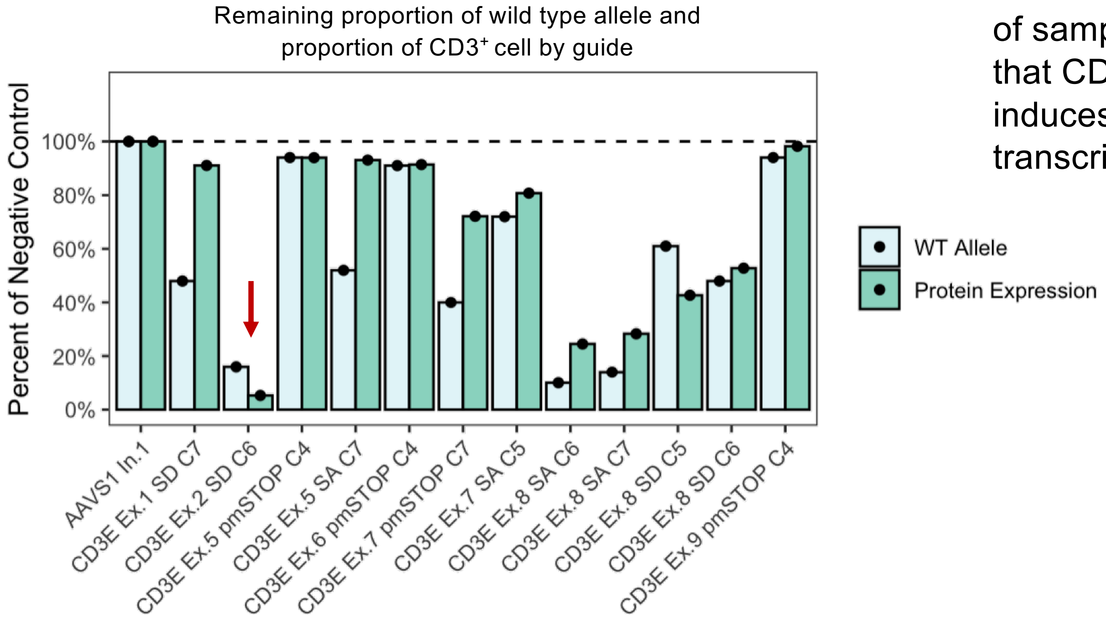

Supplementary Figure S17

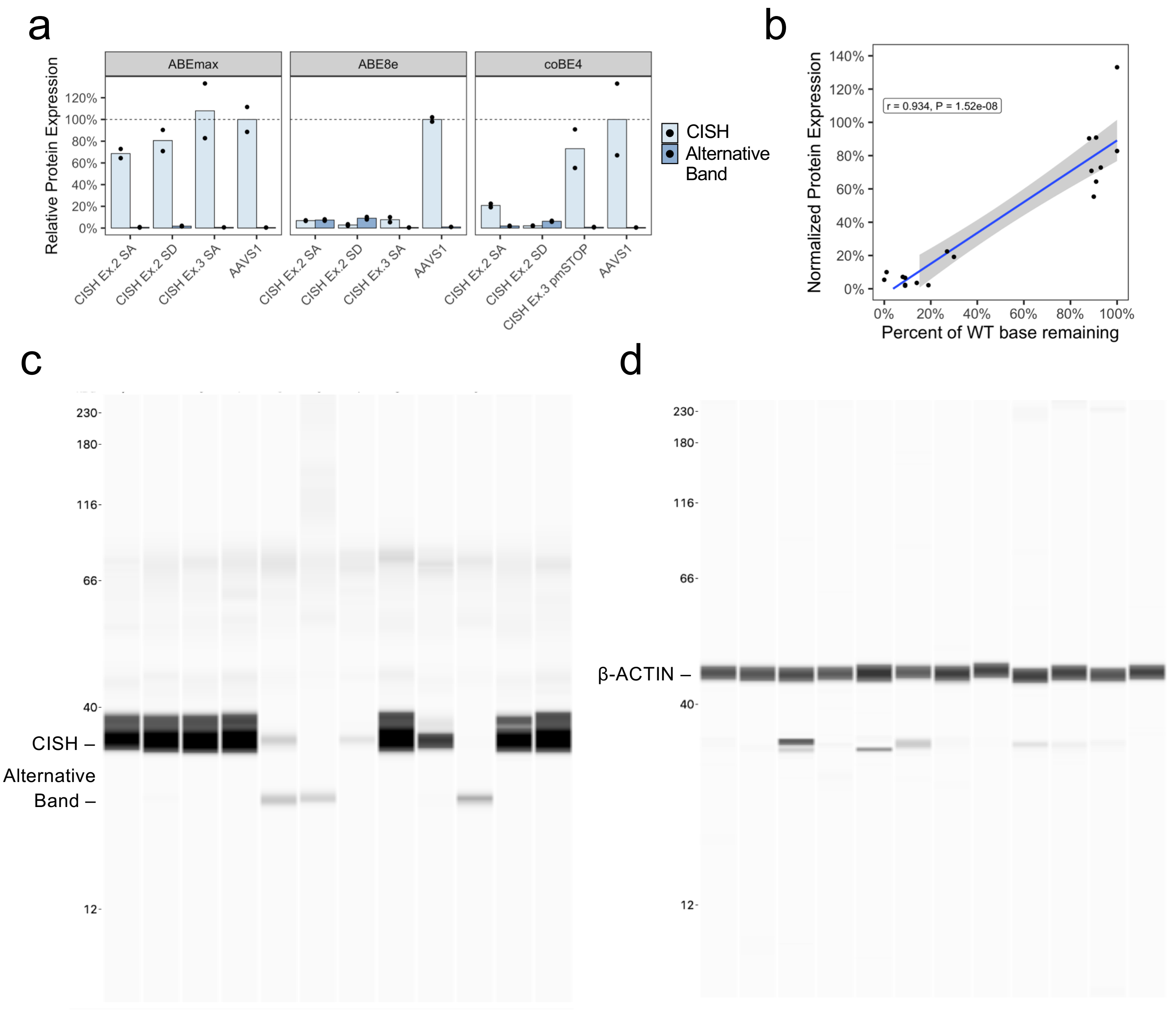

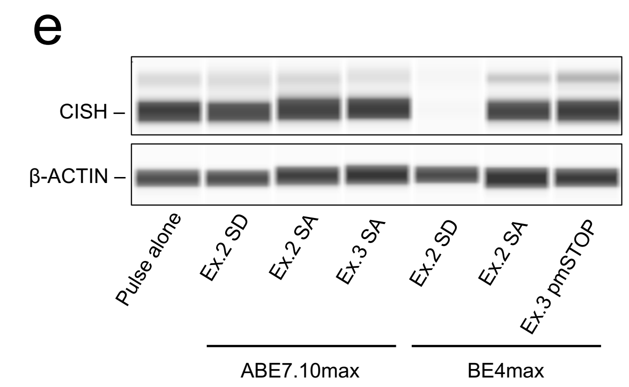

Supplementary Figure S17. Digital western blots for CISH following treatment with base editors. (a) Quantification of the relative expression of each band normalized to β-actin and the AAVS1 control. (b) Scatter plot of normalized protein expression vs. the remaining WT allele after treatment, showing a strong correlation between editing and protein knockout. Pearson's correlation coefficient (*r*) shown with two-tailed t-test of correlation coefficient. Error bands represent 95% CI of the mean. (c) Uncropped digital western blot for CISH. (d) Uncropped digital western blot for β-actin. (e) Pilot experiment in T cells before the development of ABE8e with CISH targeting guide. T cells show comparable CISH disruption to K562s. Representative gel of N = 2 biological replicates.

Supplementary Figure S18

a

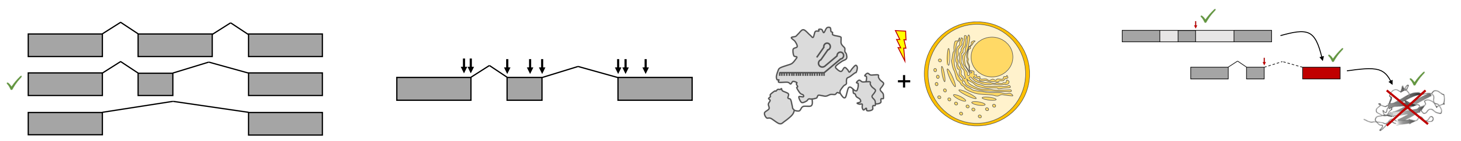

Identify Targets → **Design sgRNAs** → **Test Reagents** → **Validate Reagents**

Identify Targets

Identify Transcript
- Once a target gene is chosen, identify a target isoform
- Use the isoform with the greatest annotations on Ensembl
- Consult annotations such as CCDS, Gold Biotype, Uniprot, RefSeq, APRIS, and GENCODE
- Important to consider if isoform is expressed in cell type of interest

Identify Exons
- If possible, target exons in known functional regions
- Preferentially target exons shared across isoforms
- Uniprot annotations are useful in identifying functionally relevant regions
- E.g. active sites, docking sites, transmembrane domains, etc.
- Consult literature in target region selection

Design sgRNAs

Choosing sgRNAs
- Choose between 3-4 BE-splice sgRNAs from SpliceR with high predicted efficiency
- We regularly employ splice donor, splice acceptor, and pmSTOP sgRNAs.
- Splice donor guides have the added utility of being functional with CBEs, ABEs, and Cas9
- Assess the predicted efficiency of guides using both the Doench score[68] and BE-Hive score[40].
- Asses the risk of guide dependent off-targets (OTs) using predicted OTs

Alternative approaches
- If no guides are available, consider expanding default parameters on SpliceR
- We recommend using the iSTOP database for pmSTOP guides
- If no guides are found meeting the desired specifications, consider one of the other approaches detailed below

Test Reagents

Acquiring Reagents
- Chemically modified sgRNAs (cm-sgRNAs) synthesized with terminal phosphorthioate modifications allows for expedient testing without the need for cloning
- cm-sgRNAs are available from Synthego, IDT, TriLink, and Axolabs, among others
- Most published base editors are available as plasmids through Addgene
- Use the latest versions of base editor enzymes, e.g. ≥ BE4 or ≥ ABE8

Delivering Reagents
- Reagents can be delivered via electroporation such as with the Neon or Amaxa systems, or through lipofection.
- For applications in sensitive cell types use mRNA expressed enzyme or preformed RNP complexes to reduce cytotoxicity

Validate Reagents

DNA
- PCR amplify the region of interest from gDNA and Sanger sequence
- Use EditR to quantify editing from Sanger sequencing

RNA
- For BE-splice guides perform a whole cDNA amplification to confirm the effect of splice site disruption
- Isoforms can be sequenced

Protein
- Use a western blot or flow cytometry to validate loss of the protein product

Off-target (OT) analysis
- Employ a combination of predictive and empirical methods for assessing off-targets in tandem with NGS
- Gold standard is whole genome sequencing
- Consider employing engineered variants with reduced RNA OTs if RNA OTs are of concern

Supplementary Figure S18. (a) A standardized workflow for designing and testing base editor sgRNAs for gene disruption.

Guide Name	Gene	Protospacer	Forward Primer	Reverse Primer
B2M Ex.3 SA C6	B2M	TCGATCTATGAAAAAGACAG	TACACCTTCTTCATGCCACT	CAGTACTTTCTGGCTGGATT
B2M Ex.4 SA C4	B2M	AACCTGAAAAGAAAAGAAAA	AGCAACCTGCTCAGATACAT	AGTCCATCATTGACCAAAAC
B2M Ex.1 SD C6	B2M	ACTCACGCTGGATAGCCTCC	CACCAAGGAGAACTTGGA	CTCTAAGAAAAGGAAACTGAAAA
B2M Ex.2 pmSTOP C4	B2M	ACCCAGACACATAGCAATTC	TTCAGCAGCTTACAAAAGAA	AGAAATCGATGACCAAATGT
B2M Ex.2 pmSTOP C6	B2M	TTACCCCACTTAACTATCTT	GCATCAGTATCTCAGCAGGT	GCTATGTGTCTGGGTTTCAT
CD3D Ex.3 SA C7	CD3D	GGCACACTGTGGGGGAAGGG	AGCTCACTGGTACACACACA	TCCCAAAAGCTGAGATTACT
CD3D Ex.1 SD C8	CD3D	AGCCTTACCTTGCGAGAGAA	GCCTGGCTAATTCTTTTGTT	AAAGCAGAGAAGCAGACATC
CD3D Ex.3 pmSTOP C5	CD3D	GTGCCAGAGCTGTGTGGAGC	TCCCAAAAGCTGAGATTACT	AGCTCACTGGTACACACACA
CD3D Ex.4 pmSTOP C6	CD3D	TCTATCAGGTGAGCGTTGAG	TGGGAGTTACTCTTGCAATC	GGGCTAAGAGAGGAGAAGAG
CD3D Ex.5 pmSTOP C7	CD3D	GATGCTCAGTACAGCCACCT	GGATAGAGAGGCTCACACTG	TGAAGGAAGAAACAGGTAGG
CD3D Ex.5 pmSTOP C6	CD3D	GAGCCCAGTTTCCTCCAAGG	TGAAGGAAGAAACAGGTAGG	GGATAGAGAGGCTCACACTG
CD3D Ex.5 pmSTOP C5	CD3D	GAGCCCAGTTTCCTCCAAGG	TGAAGGAAGAAACAGGTAGG	GGATAGAGAGGCTCACACTG
CD3G Ex.3 SA C2	CD3G	TCCTGAAATGAGAAAAGCCG	ACACTTGGAGTGGTTTTGAC	CCCGAGAGCATGTTAGTAAT
CD3G Ex.3 SD C6	CD3G	ACATACTTCTGTAATACACT	GGACTGGCTGTAGTTTTTCA	CAGGAAACCACTTGGTTAAG
CD3G Ex.5 SD C10	CD3G	ATCCCCTTACCTGGTAGAGC	GTGTCCCTCTCAGTGTGTTC	GCTTCTTTCACTCAACAACA
CD3G Ex.1 pmSTOP C4	CD3G	CTTCAAGGTAAGGGCCTACT	GTTCTTGCCTTCTCTCAAAG	GTCCTCTCTTCAGCCATTTA
CD3G Ex.2 pmSTOP C6	CD3G	TGGCCCAGTCAATCAAAGGT	GGATACCAGGACAAAGATGA	GGAACAGGTTATGTCAGGTC
CD3G Ex.5 pmSTOP C6	CD3G	ATGACCAGCTCTACCAGGTA	GCTTCTTTCACTCAACAACA	GTGTCCCTCTCAGTGTGTTC
CD3G Ex.6 pmSTOP C4	CD3G	GACCAGTACAGCCACCTTCA	GAAAGACTCCGTCTCAAAAA	AGAAAAGAGTACCCCAAACC
CD3G Ex.6 pmSTOP C6	CD3G	ACCTTCAAGGAAACCAGTTG	CTCCATCTCCTTGTCCTCTT	TTACCTAGGCCAAGACAATC
CD3E Ex.5 SA C7	CD3E	TACCACCTGAAAATGAAAAA	AAACCCACAGAAGTCCCTAT	TGGTATATTCTGAATCCGATG
CD3E Ex.8 SA C7	CD3E	TTTGTCCTGCGGAGGAAGGA	CCTGTTTTACCATGAAGGAC	AGCTGAGGAAAGTCACAAAA
CD3E Ex.7 SA C5	CD3E	CACACTGTGGGGGGTGGGGT	GGAATCTCCTCTGACTGGA	ATAAAGCTGGGACTCAAACC
CD3E Ex.8 SA C6	CD3E	TTGTCCTGCGGAGGAAGGAG	CCTGTTTTACCATGAAGGAC	AGCTGAGGAAAGTCACAAAA
CD3E Ex.8 SD C5	CD3E	GTTACCTCATAGTCTGGGTT	GGAGTATGTCTCCTGCAAGC	AAAGGACGTCTGAACAGAAA
CD3E Ex.1 SD C7	CD3E	GACTCACCATTTTCTGAAGC	CATCTTTGTTTCATGGGACT	CATGGTCTGGACAGCTAAAT
CD3E Ex.8 SD C6	CD3E	CGTTACCTCATAGTCTGGGT	GGAGTATGTCTCCTGCAAGC	AAAGGACGTCTGAACAGAAA
CD3E Ex.2 SD C6	CD3E	ACTCACCTGATAAGAGGCAG	TAGCTATGATCACCCCAACT	CTTTTTCATCCTAGCATTGG
CD3E Ex.5 pmSTOP C4	CD3E	ACACAGACACGTGAGTTTAT	TCATAGGCTAAGCATGAACC	CTTTTGAGAGGTGGCTTTAG
CD3E Ex.6 pmSTOP C4	CD3E	TGCCATAGTATTTCAGATCC	TTGCCCTCAGGTAGAGATAA	CCACATATCTCCTCTTCCAC
CD3E Ex.7 pmSTOP C7	CD3E	GTGACACGAGGAGCGGGTGC	CCTTGTGTTTTCCTTGCTTA	ACTTTTCTAGGATGGGAAGG
CD3E Ex.9 pmSTOP C4	CD3E	GGCCAGCGGGACCTGTATTC	ACCAGCTCAAGTCTCCTACC	GGAAGCAGCAATATTTTAGG
CD247 Ex.6 SA C6	CD247	AGTTCCTGCAGAAGAGGGCG	ATGAGAAGTGGATGGGAAA	CCTCAGCTCAGTGCAAAG
CD247 Ex.3 SD C10	CD247	GCTGACTTACGTTATAGAGC	TTCCATGCATCAAGACATTA	CATTGTTAGTTGCCAAGGAG
CD247 Ex.1 pmSTOP C7	CD247	CAGGCACAGTTGCCGATTAC	CAGTACTTTCTGGCTGGATT	TACACCTTCTTCATGCCACT
CD247 Ex.1 pmSTOP C7	CD247	CAGGCACAGTTGCCGATTAC	CAGGTGCTTTCTCAAAGG	TATTCTAGCAGCTGGCTCTG
TRAC Ex.3 SA C8	TRAC	TTCGTATCTGTAAAACCAAG	TCTCAGAGCTTAGGATGCAC	CCAAGTCTAGTCGGTGTTTC
TRAC Ex.1 SD C5	TRAC	CTTACCTGGGCTGGGGAAGA	CACCTTCTCTTCATCTGCTT	CCTATTCACCGATTTTGATT
TRAC Ex.3 pmSTOP C4	TRAC	TTTCAAAACCTGTCAGTGAT	CTGGAAGAATGCACAGAATC	CTCAGGCTTTGGACTTAAAA
B2M Ex.2 SA A4	B2M	CTCAGGTACTCCAAAGATTC	CGGTTTATTCTTCAAAATGG	GCAGGCATACTCATCTTTTT
B2M Ex.2 SD A4	B2M	CTTACCCCACTTAACTATCT	GCATCAGTATCTCAGCAGGT	GCTATGTGTCTGGGTTTCAT
B2M Ex.1 SD A5	B2M	ACTCACGCTGGATAGCCTCC	CACCAAGGAGAACTTGGA	CTCTAAGAAAAGGAAACTGAAAA
B2M Ex.2 SD A3	B2M	TTACCCCACTTAACTATCTT	GCATCAGTATCTCAGCAGGT	GCTATGTGTCTGGGTTTCAT
CD3D Ex.3 SA A4	CD3D	CACAGTGTGCCAGAGCTGTG	TCCCAAAAGCTGAGATTACT	AGCTCACTGGTACACACACA
CD3D Ex.1 SD A7	CD3D	AGCCTTACCTTGCGAGAGAA	GCCTGGCTAATTCTTTTGTT	AAAGCAGAGAAGCAGACATC
CD3G Ex.3 SA A4	CD3G	TTCAGGAAACCACTTGGTTA	CCCGAGAGCATGTTAGTAAT	ACACTTGGAGTGGTTTTGAC
CD3G Ex.3 SD A5	CD3G	ACATACTTCTGTAATACACT	GGACTGGCTGTAGTTTTTCA	CAGGAAACCACTTGGTTAAG
CD3G Ex.5 SD A9	CD3G	ATCCCCTTACCTGGTAGAGC	GTGTCCCTCTCAGTGTGTTC	GCTTCTTTCACTCAACAACA
CD3E Ex.4 SA A7	CD3E	CTTTTCAGGTAATGAAGAAA	TTATGCCAGCCTAGATTCC	GACATGACAGCTAGCAACAA
CD3E Ex.4 SA A6	CD3E	TTTTCAGGTAATGAAGAAAT	CCATGCTGCCTAATTTGTA	GACATGACAGCTAGCAACAA
CD3E Ex.8 SA A5	CD3E	CCGCAGGACAAAACAAGGAG	AGCTGAGGAAAGTCACAAAA	CCTGTTTTACCATGAAGGAC
CD3E Ex.8 SD A4	CD3E	GTTACCTCATAGTCTGGGTT	GGAGTATGTCTCCTGCAAGC	AAAGGACGTCTGAACAGAAA
CD3E Ex.1 SD A6	CD3E	GACTCACCATTTTCTGAAGC	CATCTTTGTTTCATGGGACT	CATGGTCTGGACAGCTAAAT
CD3E Ex.8 SD A5	CD3E	CGTTACCTCATAGTCTGGGT	GGAGTATGTCTCCTGCAAGC	AAAGGACGTCTGAACAGAAA
CD3E Ex.2 SD A5	CD3E	ACTCACCTGATAAGAGGCAG	TAGCTATGATCACCCCAACT	CTTTTTCATCCTAGCATTGG
TRAC Ex.1 SD A4	TRAC	CTTACCTGGGCTGGGGAAGA	CACCTTCTCTTCATCTGCTT	CCTATTCACCGATTTTGATT
CD247 Ex.3 SA A9	CD247	CTGTTATAGGAGCTCAATCT	GTCATGTTAAGGCGTGTTCT	GGGTCTCCATCTCTTCTCTT
CD247 Ex.4 SD A8	CD247	GCTGACTTACGTTATAGAGC	TTCCATGCATCAAGACATTA	CATTGTTAGTTGCCAAGGAG
CD3G Ex.1 Cas9	CD3G	ACTGACATGGAACAGGGGAA	GTTCTTGCCTTCTCTCAAAG	GTCCTCTCTTCAGCCATTTA
CD3G Ex.3 Cas9	CD3G	AGTCATACACCTTAACCAAG	ACACTTGGAGTGGTTTTGAC	CCCGAGAGCATGTTAGTAAT
CD3D Ex.1 Cas9	CD3D	AGCCTTACCTTGCGAGAGAA	GCCTGGCTAATTCTTTTGTT	AAAGCAGAGAAGCAGACATC
CD3D Ex.3 Cas9	CD3D	GGAGCTGGATCCAGCCACCG	TCCCAAAAGCTGAGATTACT	AGCTCACTGGTACACACACA
CD3E Ex.3 Cas9	CD3E	ATTTTCTAGTTGGCGTTTGG	TTTTTCTGACTCCTGCTTGT	CTTGTGAGGAAAGTCAAAGG
CD3E Ex.6 Cas9	CD3E	AGGGCATGTCAATATTACTG	TTGCCCTCAGGTAGAGATAA	CCACATATCTCCTCTTCCAC
TRBC1 Ex.1 Cas9	TRBC	CCACACCCAAAAGGCCACAC	CTACCAGAACCAGACAGCTC	GAGAAGAATGAACCACAGGT
TRBC1 Ex.3 Cas9	TRBC	CATAGAGGATGGTGGCAGAC	TCTCTCATGGTTCTTGACCT	AGGAGTTGTGAGGATTGAGA
TRBC2 Ex.1 Cas9	TRBC	CCACACCCAAAAGGCCACAC	CAGGACCAGACAGCTCTTAG	CATTCAGCCTCTATGCTTCT
TRBC2 Ex.3 Cas9	TRBC	CATAGAGGATGGTGGCAGAC	ATTTTTCTTCCCCTGTTTTC	CTGTGATATTCCCTGCATTT
CD247 Ex.1 Cas9	CD247	GTGGAAGGCGCTTTTCACCG	GGAGGTAGCTGCAGAATAAA	CGAAAAATCTGTACCTGGAG
CD247 Ex.2 Cas9	CD247	CACCTTCACTCTCAGGAACA	AGCTTTATCTCTTGGCACAG	TCACACTTGGAAAGAAGGAC
TRAC In.1 Cas9	TRAC	TCAGGGTTCTGGATATCTGT	TCACGAGCAGCTGGTTTCTA	CCATTCCTGAAGCAAGGAAA
TRAC Ex.1 Cas9	TRAC	TCTCTCAGCTGGTACACGGC	TCACGAGCAGCTGGTTTCTA	CCATTCCTGAAGCAAGGAAA
AAVS1	AAVS1	GTCACCAATCCTGTCCCTAG	GTCTGTGCTAGCTCTTCCAG	CCATCGTAAGCAAACCTTAG
CISH Ex.2 SA C10	CISH	GGACGAGGTCTAGAAGGCAG	TGATGACAAGTGGGGAACGA	TCCCACCAGACTACTCAGGA
CISH Ex.2 SA A5	CISH	TTCTAGACCTCGTCCTTTGC	TGATGACAAGTGGGGAACGA	TCCCACCAGACTACTCAGGA
CISH Ex.2 SD C5	CISH	CTCACCAGATTCCCGAAGGT	TGATGACAAGTGGGGAACGA	TCCCACCAGACTACTCAGGA
CISH Ex.2 SD A4	CISH	CTCACCAGATTCCCGAAGGT	TGATGACAAGTGGGGAACGA	TCCCACCAGACTACTCAGGA
CISH Ex.3 SA A9	CISH	ACTTGCCTAGGCTGGTATTG	GAGAGTCTGATGGGAGAGGC	CTTCTGCGTACAAAGGGCTG
CISH Ex.3 pmSTOP C8	CISH	TGGAACCCCAATACCAGCCT	GAGAGTCTGATGGGAGAGGC	CTTCTGCGTACAAAGGGCTG

Supplementary Figure S19

Supplementary Figure S19. Table of sgRNA protospacer sequences and corresponding primers used for PCR and Sanger Sequencing.
