# Supplementary material for: CRISPR-Cas9 cytidine and adenosine base editing of splice-sites mediates highly-efficient disruption of proteins in primary and immortalized cells
Source: Nat Commun. 2021 Apr 23;12:2437. doi: 10.1038/s41467-021-22009-2 (PMC8065034; doi:10.1038/s41467-021-22009-2)
Supplement: Supplementary file 4 — Source Data [file 41467_2021_22009_MOESM4_ESM.zip › source_data/figure_reproduction.html]

CRISPR-Cas9 cytidine and adenosine base editing of splice-sites mediates highly-efficient disruption of proteins in primary and immortalized cells


# CRISPR-Cas9 cytidine and adenosine base editing of splice-sites mediates highly-efficient disruption of proteins in primary and immortalized cells

#### Mitchell G. Kluesner†, Walker S. Lahr†, Cara-Lin Lonetree, Branden A. Smeester, Xiaohong Qiu, Nicholas J. Slipek, Patricia N. Claudio-Vázquez, Samuel P. Pitzen, Emily J. Pomeroy, Madison J. Vignes, Samantha C. Lee, Samuel P. Bingea, Aneesha A. Andrews, Beau R. Webber‡, and Branden S. Moriarity‡

#### 2/22/2021

† These authors contributed equally

‡ These authors contributed equally

Copyright (C) 2020-2021 Mitchell Kluesner (klues009@umn.edu)

This file is part of the SpliceR Project

Please only copy and/or distribute this script with proper citation of *CRISPR-Cas9 cytidine and adenosine base editing of splice-sites mediates highly-efficient disruption of proteins in primary and immortalized cells*, Kluesner & Lahr et al., 2021, Nature Communications

All data and code for reproduction of this HTML file is found in `source_data`. Specifically, the .Rmd file for generating this HTML is found in `source_data/figure_reproduction.Rmd`

# Backend

## Libraries

```
# Packages required for this analysis
library(tidyverse)
library(magrittr)
library(scales)
library(gridExtra)
library(googlesheets)
library(ggseqlogo)
library(DT)
library(ggbeeswarm)

# Packages used in the SpliceR web app, but aren't required for this analysis
# library(shiny)
# library(Biostrings)
# library(magrittr)
# library(stringi)
# library(dplyr)
# library(tidyr)
# library(ggplot2)
# library(grr)
# library(printr)
# library(plyr)
# library(readr)
# library(printr)
# library(rmarkdown)
# library(DT)
# library(httr)
# library(curl)
```

## Session information and package versions

```
sessionInfo()
```

```
## R version 3.6.3 (2020-02-29)
## Platform: x86_64-apple-darwin15.6.0 (64-bit)
## Running under: macOS Catalina 10.15.7
## 
## Matrix products: default
## BLAS:   /Library/Frameworks/R.framework/Versions/3.6/Resources/lib/libRblas.0.dylib
## LAPACK: /Library/Frameworks/R.framework/Versions/3.6/Resources/lib/libRlapack.dylib
## 
## locale:
## [1] en_US.UTF-8/en_US.UTF-8/en_US.UTF-8/C/en_US.UTF-8/en_US.UTF-8
## 
## attached base packages:
## [1] stats     graphics  grDevices utils     datasets  methods   base     
## 
## other attached packages:
##  [1] ggbeeswarm_0.6.0   DT_0.17            ggseqlogo_0.1      googlesheets_0.3.0
##  [5] gridExtra_2.3      scales_1.1.1       magrittr_2.0.1     forcats_0.5.1     
##  [9] stringr_1.4.0      dplyr_1.0.3        purrr_0.3.4        readr_1.4.0       
## [13] tidyr_1.1.2        tibble_3.0.6       ggplot2_3.3.3      tidyverse_1.3.0   
## 
## loaded via a namespace (and not attached):
##  [1] beeswarm_0.2.3    tidyselect_1.1.0  xfun_0.20         haven_2.3.1      
##  [5] colorspace_2.0-0  vctrs_0.3.6       generics_0.1.0    htmltools_0.5.1.1
##  [9] yaml_2.2.1        rlang_0.4.10      pillar_1.4.7      glue_1.4.2       
## [13] withr_2.4.1       DBI_1.1.1         dbplyr_2.0.0      modelr_0.1.8     
## [17] readxl_1.3.1      lifecycle_0.2.0   munsell_0.5.0     gtable_0.3.0     
## [21] cellranger_1.1.0  rvest_0.3.6       htmlwidgets_1.5.3 evaluate_0.14    
## [25] knitr_1.31        vipor_0.4.5       broom_0.7.4       Rcpp_1.0.6       
## [29] backports_1.2.1   jsonlite_1.7.2    fs_1.5.0          hms_1.0.0        
## [33] digest_0.6.27     stringi_1.5.3     grid_3.6.3        cli_2.3.0        
## [37] tools_3.6.3       crayon_1.4.0      pkgconfig_2.0.3   ellipsis_0.3.1   
## [41] xml2_1.3.2        reprex_1.0.0      lubridate_1.7.9.2 assertthat_0.2.1 
## [45] rmarkdown_2.6     httr_1.4.2        rstudioapi_0.13   R6_2.5.0         
## [49] compiler_3.6.3
```

## Global parameters

```
colors5 = c("#d7191c", "#fdae61", "khaki", "#abd9e9", "#2c7bb6")
colors = c("NGA" = "#66c2a5", "NGT" = "#fc8d62", "NGC" = "#8da0cb",
           "NGG" = "#e78ac3", "NGN" = "#b3b3b3")
gene_colors = c(B2M = "#d9d9d9", TRAC = '#8dd3c7', TRBC = '#ffffb3', CD3G = '#bebada', CD3D = '#fb8072',CD3E = '#80b1d3',CD247 = '#fdb462', AAVS1  = "#b3de69")
```

## Data paths

All files are assumed to be located within `source_data` directory

```
ensembl_ids_path = "protein.txt"
proteing_coding_guides_path = "protein_guides_1-95563_2018.09.06.tsv"
protein_coding_genes_path = "protein_genes_1-97713_2018.09.06.tsv"

experimental_data_path = "experimental_data.tsv"
cbe_meta_data_path = "CBE_meta_data.tsv"
abe_meta_data_path = "ABE_meta_data.tsv"

context_weights_path = "context_weights.tsv"
position_weights_path = "position_weights.tsv"
motif_weights_path = "motif_weights.tsv"

be4_guides_flanked_path = "BE4_guides_flanked.tsv"
be4_guides_predicted_path = "BE4_guides_predicted.tsv"
abe_guides_flanked_path = "ABE_guides_flanked.tsv"
abe_guides_predicted_path = "ABE_guides_predicted.tsv"

CISH_path = "CISH.tsv"
CISH_taqman_path = "CISH_taqman.tsv"
```

## Define functions

```
len_uni = function(x){length(unique(x))}
as.percent = function(x, decimal_place = 4){paste0(round(x, decimal_place)*100, "%")}

correlation = function(x, y){cor.test(x = x, y = y)$estimate}
correlation_pvalue = function(x, y){cor.test(x = x, y = y)$p.value}

flushOutData = function(df, base){
  # df = abe_data
  # base = "A"
  # Take a rowise approach
  # create df_a that has the cols Protospacer, Cell type, Paper, normalization, G_ave = 0, and G_norm
  df_a = df %>% dplyr::select(Protospacer)
  
  # create df_b data frame that has the cols Position, and Protospacer for each guide
  guides = df_a$Protospacer %>% unique()
  list_a = str_locate_all(guides, base)
  
  names(list_a) = guides
  vec_b = unlist(list_a)
  
  df_b = data.frame(Protospacer = names(vec_b), Position = vec_b) %>%
    mutate(Protospacer = gsub("[0-9]", "", Protospacer)) %>%
    distinct()
  
  # df_c = inner_join(df_a, df_b)
  df_c = inner_join(df_a, df_b) %>% distinct()
  
  # df_d = remove any rows from df_c that are in the data, i.e remove the filler rows for already documented values
  df_d = anti_join(df_c, df) %>%
    mutate(Edit_ave = 0, Edit_norm = 0)
  
  # bind_cols(data, df_d)
  df_e = bind_rows(df_d, df)
  
  # recalculate the motifs
  final_data = df_e %>% 
    mutate(Trinucleotide = substr(Protospacer, Position-1, Position+1)) %>%
    mutate(Dinucleotide = substr(Protospacer, Position-1, Position)) %>%
    mutate(Dinucleotide = factor(Dinucleotide, levels = paste0(c("T", "C", "A", "G"), base))) %>%
    mutate(PostDinucleotide = substr(Protospacer, Position, Position+1)) %>%
    mutate(PostDinucleotide = factor(PostDinucleotide, levels = paste0(base, c("T", "C", "G", "A")))) %>%
    mutate(TrinucleotideClass = gsub("[A|G]", "R", Trinucleotide) %>% gsub("[T|C]", "Y", .))
  
  filler_data = data.frame(Dinucleotide = c(rep(paste0(c("A", "T", "C", "G"), base), 4), rep(paste0(c("A", "T", "C", "G"), base), 4)),
                           PostDinucleotide = c(rep(paste0(base, c("A", "T", "C", "G")), 4), rep(paste0(base, c("A", "T", "C", "G")), 4)),
                           Edit_norm = rep(rep(0, 4), 4), Position = c(rep(rep(1, 4), 4), rep(rep(0, 4), 4)))
  
  output_data = bind_rows(final_data, filler_data)
  
  final_output_data = bind_rows(
    output_data %>% mutate(Dinucleotide = "All", PostDinucleotide = "All"),
    output_data
    ) %>%
    mutate(Dinucleotide = factor(Dinucleotide, levels = c("All", paste0(c("T", "C", "A", "G"), base)))) %>%
    mutate(PostDinucleotide = factor(PostDinucleotide, levels = c("All", paste0(base, c("T", "C", "G", "A")))))
    
    
  return(final_output_data)
}

# Logo plots of each guide
plotLogo = function(data, facet, method = "bit"){
  
  plotting_data = data %>%
    mutate(TargetMotif = toupper(substr(Protospacer, start = Position - 2, stop = Position + 2))) %>%
    mutate(TargetMotif = {ifelse(nchar(TargetMotif) < 5, paste0(TargetMotif, " "), TargetMotif)}) %>% 
    inner_join(., data.frame(stringsAsFactors = F, 
                             Motif = c("SA", "SD", "pmSTOP"), Motif_Name = c("Acceptor", "Donor", "pmSTOP"))) %>%
    mutate(Facet = paste0(Enzyme, " ", Motif_Name))
           
  facets = unique(plotting_data$Facet)
  
  plotting_data %>%
    filter(Facet == facets[facet]) %>%
    filter(!is.na(TargetMotif) & nchar(TargetMotif) == 5) %>%
    dplyr::select(TargetMotif) %>%
    ggseqlogo::ggseqlogo(., method = method) +
    scale_y_continuous(limits = c(0,2), breaks = seq(0,2,0.5), labels = seq(0,2,0.5)) +
    scale_x_continuous(breaks = 1:5, labels = c("N-2", "N-1", "Target", "N+1", "N+2")) +
    ggtitle(facets[facet]) +
    theme_bw(base_size = 32) +
    theme(panel.grid = element_blank(), aspect.ratio = 1)
}

### functions
transpose_df = function(df) {
  t_df = data.table::transpose(df)
  colnames(t_df) = rownames(df)
  rownames(t_df) = colnames(df)
  t_df = t_df %>%
    tibble::rownames_to_column(.data = .) %>%
    tibble::as_tibble(.)
  return(t_df)
}

shifter = function(x, n = 1) {
  if (n == 0) x else c(tail(x, n), head(x, -n))
}

probability = function(logit){mapply(FUN = function(l){exp(l)/(1 + exp(l))}, l = logit)}
logit = function(probability){mapply(FUN = function(p){log(p/(1-p))}, p = probability)}

pullPredictions = function(data, i, base){
  # data = guides
  # i = 2
  # base = "C"
  
  position = data[i,'Position']
  
  data[i,] %>%
    dplyr::pull(predictions) %>%
    .[1] %>%
    read_csv(file = .) %>%
    dplyr::select(base, paste0(base, position)) %>%
    mutate(base = paste0("p", base)) %>%
    transpose_df() %>%
    janitor::row_to_names(1) %>%
    mutate(pA = as.numeric(pA),
           pC = as.numeric(pC),
           pG = as.numeric(pG),
           pT = as.numeric(pT)) %>%
    bind_cols(data[i,], .)
}

plotFigure4E = function(var.1, var.2){
  
  # var.1 = "BE-Hive"
  # var.2 = "Observed"
  
  var.data = meta_analysis %>%
    mutate(var.1 = logit(!! sym(var.1)), var.2 = logit(!! sym(var.2)))
  
  cor.label = var.data %$%
    cor.test(x = .$var.1 , y = .$var.2, method = "spearman", exact = FALSE) %$%
    paste0("ρ = ", signif(.$estimate, 3))
  
  var.data %>%
    ggplot(aes(x = var.1, y = var.2, color = Enzyme)) +
    geom_point() +
    scale_color_brewer(palette = "Set1") +
    geom_smooth(method = "lm", color = "black") +
    ylab(paste0("Logit of ", var.2))+
    xlab(paste0("Logit of ", var.1))+
    annotate("text", -Inf, Inf, label = cor.label, hjust = -0.2, vjust = 2, size = 6) +
    theme_bw(base_size = 18) +
    ggtitle(paste0(var.1, " vs ", var.2)) +
    theme(panel.grid = element_blank(),
          aspect.ratio = 1
    )
}

supplementaryFigure2and3 = function(data_tmp, enzyme, motif, figure){
  # enzyme = "ABE"
  # motif = "acceptor"
  
  ## Establish temporary dataframe
  message("filtering data.")
  figure_data_tmp = data_tmp %>%
    # filter on desired enzyme
    filter(grepl(enzyme, be)) %>%
    # filter on desired splice_site
    filter(splice_site == motif) %>%
    # duplicate the data for NGN pam for aggregating and plotting purposes
    mutate(NGN = "NGN") %>%
    gather(type, PAM, c("pam_class", "NGN")) %>%
    group_by(gene_id, splice_site, PAM) %>%
    # summarize the earliest guide and number of guides for each gene for each pam
    dplyr::summarise(earliest_guide = min(position_score), number_of_guides = length(position_score)) %>%
    # factor for plotting order
    mutate(PAM = factor(PAM, levels = c("NGG", "NGA", "NGT", "NGC", "NGN")))
  
  # Change enzyme name for plotting
  enzyme = if(enzyme == "ABE") {"ABE"} else {"CBE"}
  
  ## Plotting
  # define the axis adjustment scalar
  axis_adjustment = sum(nsesi_gene_ids %in% (figure_data_tmp %>% .$gene_id))/length(nsesi_gene_ids)
  # define the earliest percent distance into the transcript 50% of the guides have
  guide50_tmp = figure_data_tmp %>% filter(PAM == "NGN") %>% .$earliest_guide %>% median
  # define the number of guides 50% of genes have
  number50_tmp = figure_data_tmp %>% filter(PAM == "NGN") %>% .$number_of_guides %>% median
  
  # Plot the cumulative earliest guide position
  message("making earliest guide plot")
  
  figureS3 = figure_data_tmp %>%
    ggplot(aes(x = earliest_guide, color = PAM)) +
    stat_ecdf(geom = "step", pad = FALSE, lwd = 1.5, alpha = 0.8) +
    scale_y_continuous(limits = c(0,1)/axis_adjustment, breaks = seq(0,1,0.2)/axis_adjustment, labels = paste0(seq(0,1,0.2)*100, "%")) +
    scale_x_continuous(limits = c(0,1), breaks = seq(0, 1, 0.2)) +
    ggtitle(paste0(enzyme, " splice ", motif, " sgRNAs")) +
    ylab("Cumulative % of targetable genes") + 
    xlab("Earliest BE-Splice targetable splice-site\n(relative position in mRNA)") +
    coord_cartesian(xlim = c(0, 1.0), ylim = c(0, 1)/axis_adjustment, expand = F) +
    theme_bw(base_size = 24) +
    theme(panel.border = element_rect(colour = "black", fill=NA),
          aspect.ratio = 1,
          legend.position = c(0.825,  0.25/axis_adjustment),
          legend.background = element_rect(fill = "white", color = "black"),
          plot.margin = unit(c(1,1,1,1), "cm")
    ) +
    labs(color = "PAM") +
    scale_color_manual(values = colors) +
    geom_hline(yintercept = axis_adjustment/axis_adjustment, linetype = "dashed") +
    annotate(geom = "text", size = 5, x = if(motif == "acceptor"){0.2}else{0.8}, y = 0.90, label = paste0(as.percent(axis_adjustment)," of genes\nare targettable")) +
    annotate(geom = "text", size = 5, x = if(motif == "acceptor"){0.2}else{0.8}, y = 0.625, label = paste0("50% of earliest guides\nare ", as.percent(guide50_tmp), " into the\ntranscript or earlier")) +
    annotate(geom = "segment",  x = guide50_tmp, xend = guide50_tmp, y = 0, yend = 0.5, linetype = "dashed") +
    annotate(geom = "segment",  x = 0, xend = guide50_tmp, y = 0.5, yend = 0.5, linetype = "dashed")
  ggsave(paste0("/Users/kluesner/Desktop/Research/spliceR/submissions/figures/figureS2/", enzyme, "_", motif, "_earliestGuide.tiff"), device = "tiff")
  
  figureS2 = figure_data_tmp %>%
    ggplot(aes(x = number_of_guides, color = PAM)) +
    stat_ecdf(geom = "step", pad = FALSE, lwd = 1.5, alpha = 0.9) +
    scale_y_continuous(limits = c(0,1)/axis_adjustment, breaks = seq(0,1,0.2)/axis_adjustment, labels = paste0(seq(0,1,0.2)*100, "%")) +
    scale_x_log10() +
    annotation_logticks(sides = "b") +
    ylab("Cumulative % of targetable genes") + 
    xlab("Number of BE-Splice gRNAs per gene") +
    ggtitle(paste0(enzyme, " splice ", motif, " sgRNAs")) +
    coord_cartesian(xlim = c(0.9999, max(figure_data_tmp$number_of_guides)), ylim = c(-0.005, 1.005/axis_adjustment), expand = F) +
    theme_bw(base_size = 24) +
    theme(panel.border = element_rect(colour = "black", fill=NA),
          aspect.ratio = 1,
          legend.position = c(0.8,  0.25/axis_adjustment),
          legend.background = element_rect(fill = "white", color = "black"),
          plot.margin = unit(c(1,1,1,1), "cm")
    ) +
    labs(color = "PAM") +
    scale_color_manual(values = colors) +
    geom_hline(yintercept = axis_adjustment/axis_adjustment, linetype = "dashed") +
    annotate(geom = "text", size = 5, x = 10^(0.8*log10(max(figure_data_tmp$number_of_guides))), y = 0.90, label = paste0(as.percent(axis_adjustment)," of genes\nare targettable")) +
    annotate(geom = "text", size = 5, x = 10^(0.8*log10(max(figure_data_tmp$number_of_guides))), y = 0.625, label = paste0("50% of targeted genes\nhave ", number50_tmp, " guides or more\ntargeting them")) +
    annotate(geom = "segment",  x = number50_tmp, xend = number50_tmp, y = 0, yend = 0.5, linetype = "dashed") +
    annotate(geom = "segment",  x = 0, xend = number50_tmp, y = 0.5, yend = 0.5, linetype = "dashed")
  
  if(figure == "S2") {return(figureS2)} else {return(figureS3)}
}
```

## Load data

```
### Ensembl transcript IDs
ids = read_tsv(ensembl_ids_path)$ENST

### Human protein coding gene guides
guides = read_tsv(proteing_coding_guides_path) %>%
  # Convert the position_score to position in the molecule
  mutate(position_score = 1-position_score) %>%
  # Remove any empty entries that  did not produce any guides
  filter(!is.na(be)) %>%
  # Remove the version number from the Ensembl transcript ID
  # to be paired with other dataset in later analysis
  tidyr::separate(., col = id, into = c("tmp_id"), sep = '[.]', remove = FALSE) %>%
  # convert BE to include if it is targetable by BE3 or ABE
  mutate(be = {ifelse(splice_site == "donor", "BE3 or ABE", be)}) %>%
  # Make a generic PAM class for each pam
  mutate(pam_class = sub("^.", "N", pam))

### Establish genes used for guide screen
genes = read_tsv(protein_coding_genes_path) %>%
  # Remove the version number from the Ensembl transcript ID to be paired with other dataset in later analysis
  tidyr::separate(., col = id, into = c("tmp_id"), sep = '[.]', remove = FALSE)

### Experimental data
experimental_data = read_tsv(experimental_data_path) %>%
  mutate(Indel = Indel/100) %>%
  mutate(Edit = {ifelse(Enzyme == "BE4", `T`, `G`)/100}) %>%
  mutate(Protein_Loss = 1-Flow) %>%
  mutate(Gene = factor(Gene, levels =  c("TRAC", "TRBC", "CD3D", "CD3E", "CD3G", "CD247", "B2M", "AAVS1"))) %>%
  mutate(Edit = {ifelse(Enzyme == "Cas9", Indel, Edit)}) %>%
  mutate(Enzyme_Motif = paste0(Enzyme, " ", Motif)) %>%
  mutate(Enzyme_Motif = factor(Enzyme_Motif, levels = c("BE4 SD", "BE4 SA", "BE4 pmSTOP", "ABE7.10 SD", "ABE7.10 SA", "Cas9 Control"))) %>%
  mutate(Exon_Class = {ifelse(Exon == 1, "First", 
                              ifelse(Exon == 2, "Second",
                                     ifelse(Exon == N_Exons, "Last", 
                                            ifelse(Exon == N_Exons - 1, "Second-to-last", "Middle"))))}) %>%
  mutate(Exon_Class = factor(Exon_Class, levels = c("First", "Second", "Middle", "Second-to-last", "Last"))) %>%
  mutate(Exon_Group = {ifelse(Exon < N_Exons - 1, Exon, 
                              ifelse(Exon == N_Exons - 1, "Second-to-last",
                                     ifelse(Exon == N_Exons, "Last", "NA")))}) %>%
  mutate(Exon_Group = factor(Exon_Group, levels = c(1:7, "Second-to-last", "Last"))) %>%
  mutate(Enzyme = factor(Enzyme, levels = c("BE4", "ABE7.10", "Cas9"))) %>%
  mutate(Motif = factor(Motif, levels = c("SA", "SD", "pmSTOP")))

### CBE base editing by position
cbe_data = read_tsv(cbe_meta_data_path) %>%
  
  mutate(Protospacer = toupper(Protospacer)) %>%
  mutate(Edit = `T`) %>%
  
  # Establish the maximum edit for each cell type in each paper
  group_by(`Cell type`, Paper) %>%
  mutate(paper_max = max(Edit)) %>%
  ungroup %>%
  
  # Normalize the edits to the max edit observed in the paper
  mutate(Edit_norm = Edit/paper_max) %>%
  
  # For each position in each unique guide, calculate the average normalized edit
  group_by(Position, Protospacer) %>%
  dplyr::summarise(Edit_norm = mean(Edit_norm)) %>%
  ungroup() %>%
  arrange(Protospacer)


### ABE base editing by position
abe_data = read_tsv(abe_meta_data_path) %>%
  
  mutate(Protospacer = toupper(Protospacer)) %>%
  mutate(Edit = `G`) %>%
  
  # Establish the maximum edit for each cell type in each paper
  group_by(`Cell type`, Paper) %>%
  mutate(paper_max = max(Edit)) %>%
  ungroup %>%
  
  # Normalize the edits to the max edit observed in the paper
  mutate(Edit_norm = Edit/paper_max) %>%
  
  # For each position in each unique guide, calculate the average normalized edit
  group_by(Position, Protospacer) %>%
  dplyr::summarise(Edit_norm = mean(Edit_norm)) %>%
  ungroup() %>%
  arrange(Protospacer)

context_weights = read_tsv(context_weights_path )
position_weights = read_tsv(position_weights_path)
motif_weights = read_tsv(motif_weights_path)

# BE4 data
# Load in th empirical data 
BE4_guides = read_tsv(be4_guides_flanked_path) %>%
  mutate(Protospacer = toupper(Protospacer))

# Load in the predicted data
BE4_guides_predicted = read_tsv(be4_guides_predicted_path) %>%
  mutate(Protospacer = toupper(Protospacer))

#####  ABE data
ABE_guides = read_tsv(abe_guides_flanked_path) %>%
  mutate(Protospacer = toupper(Protospacer))

ABE_guides_predicted = read_tsv(abe_guides_predicted_path) %>%
  mutate(Protospacer = toupper(Protospacer))

### CISH genetic and protein data
CISH = read_tsv(CISH_path) %>%
  mutate(Enzyme = gsub("ABEmax", "ABE7.10", Enzyme)) %>%
  mutate(Enzyme = factor(Enzyme, levels = c("ABE7.10","ABE8e","coBE4"))) %>%
  mutate(Edit = Edit/100) %>%
  mutate(Guide = factor(Guide, levels = c("CISH Ex.2 SA", "CISH Ex.2 SD", "CISH Ex.3 SA", "CISH Ex.3 pmSTOP", "AAVS1")))

### CISH Taqman data
CISH_taqman = read_tsv(CISH_taqman_path) %>%
  mutate(Enzyme = gsub("ABEmax", "ABE7.10", Enzyme)) %>%
  mutate(Enzyme = factor(Enzyme, levels = c("ABE7.10", "ABE8e", "coBE4"))) %>%
  mutate(Guide = factor(Guide, levels = c("CISH Ex.2 SA", "CISH Ex.2 SD", "CISH Ex.3 SA", "CISH Ex.3 pmSTOP", "AAVS1"))) %>%
  rowwise() %>%
  mutate(ave_Cq = mean(c(Cq1, Cq2, Cq3), na.rm = TRUE)) %>%
  ungroup()
```

# Figures

## Figure 1

### Legend

Figure 1. Overview of the BE-splice approach. (a) Generalized base editor mechanism of CBEs and ABEs. (b) Positioning of BE-splice sgRNAs within conserved splice donor and splice acceptors motif. Logo plots were generated from all human protein coding gene splice sites. Arrows indicate the base targeted by either CBEs (blue), or ABEs (green). (c) Breakdown of transcripts and genes targetable by BE-splice, showing the vast majority of spliced genes are targetable by this approach (99.68%). (d) Distribution of BE-splice sgRNA density across each gene. 50% of genes have 62 or more sgRNAs mapping to them when accounting for all PAM identities and both CBE and ABE approaches. (e) Distribution of the position of the first sgRNA for each gene, with 50% having their first sgRNA 11.13% way through the mRNA or earlier. Source data and code are available in the Source Data file.

Establish the descriptive statistics for the analysis

```
### Establish various IDs for different parts of the analysis

# Ensembl gene IDs
gene_ids = genes$gene_id %>% unique()

# Ensembl transcript IDs a that are non-single exon, single isoform
nsesi_ids = genes %>%
  group_by(id) %>%
  summarize(number = length(id)) %>%
  filter(number > 1) %>%
  .$id

# Ensembl gene IDs a that are non-single exon, single isoform
nsesi_gene_ids = genes %>%
  group_by(gene_id) %>%
  summarize(number = length(gene_id)) %>%
  filter(number > 1) %>%
  .$gene_id

figure_1_data = guides %>%
  mutate(NGN = "NGN")  %>%
  gather(type, PAM, c("pam_class", "NGN")) %>%
  group_by(gene_id, PAM) %>%
  dplyr::summarise(earliest_guide = min(position_score), number_of_guides = length(position_score)) %>%
  mutate(PAM = factor(PAM, levels = c("NGG", "NGA", "NGT", "NGC", "NGN")))

guide50 = figure_1_data %>% filter(PAM == "NGN") %>% .$earliest_guide %>% median
number50 = figure_1_data %>% filter(PAM == "NGN") %>% .$number_of_guides %>% median


## Basic stats
    # Percent of all transcript ids that are non-single-exon-single-isoform (NSESI)
    perc_nsesi_ids = length(nsesi_ids)/length(ids)
    
    # Percent of all gene ids that are NSESI
    perc_nsesi_gene_ids = length(nsesi_gene_ids)/length(gene_ids)
    
    ## Transcripts
    # Number of transcript ids that yielded a gRNA that allows it to be targetable
    n_targettable_ids = (ids %in% guides$id) %>% sum()
    
    # Percent of total transcript ids that are targetable
    perc_targettable_ids = n_targettable_ids/length(ids)
    
    # Number of NSESI transcript ids of all of the ensembl transcript ids
    n_nsesi_targettable_ids = (nsesi_ids %in% guides$id) %>% sum()
    
    # Percent of NSESI transcript ids that are targetable (i.e. have potential guides)
    perc_nsesi_targettable_ids = n_targettable_ids/length(nsesi_ids)
    
    ## Genes
    # Number of genes that were found to have guides to them, i.e. targetable
    n_targettable_genes = gene_ids %in% guides$gene_id %>% sum()
    
    # percent of all genes that were found to have a gRNA to them
    perc_targettable_genes = n_targettable_genes/length(gene_ids)
    
    # Number of ensembl gene ids that are NSESI
    n_nsesi_targettable_genes = nsesi_gene_ids %in% guides$gene_id %>% sum()
    
    # Percent of NSESI gene ids that were found to have a gRNA to them
    perc_nsesi_targettable_genes = n_nsesi_targettable_genes/length(nsesi_gene_ids)
```

### Figure 1A

Diagram of base editing mechanism

### Figure 1B

Diagram of stereotypical splice donor and splice acceptor motifs with mapped guides

### Figure 1C

Table of available BE-splice sgRNAs for human protein coding genes

```
# Cumulative percent of targetable genes a sa function of the number of BE-splice gRNAs per gene
# Scoring was defined based on Komor et al., 2016 and Gaudelli et al., 2017
figure_1C = tibble(`Protein Coding` = c("Transcripts", "Genes"), 
                   Number = c(n_targettable_ids, n_targettable_genes),
                   `Percent of Spliced Genes` = c(perc_nsesi_targettable_ids, perc_nsesi_targettable_genes) %>% signif(4) %>% scales::percent(),
                   `Percent of Total` = c(perc_targettable_ids, perc_targettable_genes) %>% signif(4) %>% scales::percent()
)

figure_1C %>%
  datatable()
```

### Figure 1D

Cumulative number of BE-splice sgRNAs per gene

```
figure_1_data %>%
  ggplot(aes(x = number_of_guides, color = PAM)) +
  stat_ecdf(geom = "step", pad = FALSE, lwd = 1.5, alpha = 0.9) +
  scale_y_continuous(labels = scales::percent_format(), limits = c(0, 1)) +
  scale_x_log10() +
  annotation_logticks(sides = "b") +
  ylab("Cumulative % of targetable genes") + 
  xlab("Number of BE-Splice gRNAs per gene") +
  coord_cartesian(xlim = c(0.9999, max(figure_1_data$number_of_guides)), ylim = c(-0.005, 1.005), expand = F) +
  theme_bw(base_size = 24) +
  theme(panel.border = element_rect(colour = "black", fill=NA),
        aspect.ratio = 1,
        legend.position = c(0.825,  0.6),
        legend.background = element_rect(fill = "white", color = "black"),
        plot.margin = unit(c(1,1,1,1), "cm")) +
  labs(color = "PAM") +
  scale_color_manual(values = colors) +
  annotate(geom = "segment",  x = number50, xend = number50, y = 0, yend = 0.5, lwd = 1, linetype = "dashed") +
  annotate(geom = "segment",  x = 0, xend = number50, y = 0.5, yend = 0.5, lwd = 1, linetype = "dashed") +
  annotate(geom = "text", size = 8, x = 10^(0.14*log10(max(figure_1_data$number_of_guides))), y = 0.55, label = paste0(number50, " guides"))
```

### Figure 1E

Cumulative percent of targettable genes as a function of the earliest targettable splice-site

```
figure_1_data %>%
  ggplot(aes(x = earliest_guide, color = PAM)) +
  stat_ecdf(geom = "step", pad = FALSE, lwd = 1.5, alpha = 0.8) +
  scale_y_continuous(labels = scales::percent_format(), limits = c(0, 1)) +
  scale_x_continuous(limits = c(0,1.2), breaks = seq(0, 1.2, 0.2)) +
  ylab("Cumulative % of targetable genes") + 
  xlab("Earliest BE-Splice targetable splice-site\n(relative position in mRNA)") +
  coord_cartesian(xlim = c(0, 1.0), ylim = c(0, 1), expand = F) +
  theme_bw(base_size = 24) +
  theme(panel.border = element_rect(colour = "black", fill=NA),
        aspect.ratio = 1,
        legend.position = c(0.825,  0.6),
        legend.background = element_rect(fill = "white", color = "black"),
        plot.margin = unit(c(1,1,1,1), "cm")) +
  labs(color = "PAM") +
  scale_color_manual(values = colors) +
  annotate(geom = "segment",  x = guide50, xend = guide50, y = 0, yend = 0.5, lwd = 1, linetype = "dashed") +
  annotate(geom = "segment",  x = 0, xend = guide50, y = 0.5, yend = 0.5, lwd = 1, linetype = "dashed") +
  annotate(geom = "text", size = 8, x = 0.3, y = 0.44, label = paste0(as.percent(guide50), " into\nmRNA"))
```

## Figure 2

### Legend

Figure 2. Conception and validation of the TCR-CD3-MHC Class I immune synapse as a screening model for functional knock-out. (a) Diagram of the multimeric TCR-CD3 complex and MHC Class I immune synapse containing multiple spliced genes, based on the solved structures (PDB 6JXR43, PDB 3T0E47; PDB 10GA48). (b) Diagram of the synthesis and localization of the TCR-CD3 complex and interaction with MHC Class I. All members of the CD3 complex are required before functional localization to the cell surface, where disruption of a single splice site within one gene member can prevent a surface expressed complex from forming. (c) Cas9 nuclease knock-out of each individual member of TCR-CD3 complex validates the screening model. Two Cas9 nuclease sgRNAs were designed to exonic regions of each gene in the complex. All genes had at least one guide with >85% indel efficiency and loss in TCR-CD3 surface expression. Height of bars represents mean of N = 2 independent donors. Source data and code are available in the Source Data file.

### Figure 2A

TCR-CD3 MHC class I immunosynapse

### Figure 2B

Trafficking of TCR-CD3 MHC Class I to T cell surface

### Figure 2C

Cas9 mediated disruption of TCR-CD3 complex

```
experimental_data %>%
  filter(Experiment == "STE2") %>%
  filter(Gene != "AAVS1") %>%
  dplyr::rename(`Protein Loss` = Protein_Loss) %>%
  gather(metric, value, c("Protein Loss", "Indel")) %>%
  ggplot(aes(x = Guide_Name, y = value, fill = Gene)) +
  geom_bar(stat = "summary", fun.y = "mean", color  = 'black') +
  geom_point() +
  scale_y_continuous(limits = c(0,1), breaks = seq(0,1,0.2), labels = scales::percent_format()) +
  scale_fill_manual(values = gene_colors) +
  facet_grid(cols = vars(Gene), rows = vars(metric), space = "free_x", scale = "free_x") +
  ylab("Percent knockout") +
  xlab("") +
  theme_bw(base_size = 18) +
  theme(axis.text.x = element_text(hjust = 1, angle = 45),
        panel.grid.major = element_blank(), panel.grid.minor = element_blank())
```

Corresponding statistics for Cas9 disruption data

```
### Figure 2 statistics
experimental_data %>%
  filter(Experiment == "STE2") %>%
  group_by(Experiment) %>%
  dplyr::summarise(mean_indel = mean(Edit), mean_protein_loss = mean(Protein_Loss),
                   sd_indel = sd(Edit), sd_protein_loss = sd(Protein_Loss)) %>%
  ungroup() %>%
  dplyr::select(-Experiment) %>%
  mutate_all(~signif(.,digits= 3) %>% scales::percent()) %>%
  mutate(Experiment = "STE2") %>%
  dplyr::select(Experiment, everything()) %>%
  datatable()
```

## Figure 3

### Legend

Figure 3. BE-splice sgRNAs mediate robust editing and disruption of TCR-CD3 MHC Class I immune synapse. (a) Editing efficiency (top) and surface protein loss (bottom) from each guide in the sgRNA screen. Results grouped by gene and enzyme used in descending order by protein loss. X-axis label indicates position of target base within sgRNA. TRBC1 and TRBC2 were omitted from the BE-splice screen due to the inability to design single BE-splice sgRNAs to target both paralogs simultaneously. All edits represent the efficiency of target editing; C:G-to-T:A for CBE, and A:T-to-G:C for ABE. Height of bars are mean of replicates. (b-c) Base editing efficiencies or protein loss efficiencies grouped by enzyme and target motif. Data analyzed with Student’s two-tailed t-test if variance was equal, or Welch’s two-tailed t-test if variance was unequal with exact P-values shown. Boxplot center lines represent the median, box limits represent the upper and lower quartiles, and whiskers define the 1.5x interquartile range. (d) Consistency of editing efficiency and protein loss across all approaches employed here. Relationship between protein loss and base editing efficiency is comparable to that observed in Cas9 control. Error bands represent 95% CI of the mean. All data is from N = 2 independent donors, performed on different days. Source data and code are available in the Source Data file.

### Figure 3A

Editing efficiency (top) and surface protein loss (bottom) from each guide in the sgRNA screen.

```
experimental_data %>%
  filter(Experiment != "STE2") %>%
  mutate(Enzyme_factor = {ifelse(Enzyme ==  "BE4", 1, 2)}) %>%
  dplyr::rename(`Protein Loss` = Protein_Loss) %>%
  gather(metric, value, c("Protein Loss", "Edit")) %>%
  ggplot(aes(x = reorder(Guide_Name, -value)  %>% reorder(., Enzyme_factor), y = value, fill = Gene, shape  = Enzyme)) +
  geom_bar(stat = "summary", fun.y = "mean", color  = 'black') +
  geom_point(size = 3, aes(color = Enzyme)) +
  scale_color_manual(values = c("BE4" = "black", "ABE7.10" = "darkred")) +
  scale_y_continuous(limits = c(0,1), breaks = seq(0,1,0.2), labels = scales::percent_format()) +
  scale_fill_manual(values = gene_colors) +
  facet_grid(cols = vars(Gene), rows = vars(metric), space = "free", scale = "free") +
  ylab("") +
  xlab("") +
  theme_bw(base_size = 24) +
  theme(axis.text.x = element_text(hjust = 1, angle = 60),
        panel.grid.major = element_blank(), panel.grid.minor = element_blank())
```

```
### Figure 3A statistics
experimental_data %>%
  filter(Experiment != "STE2") %>%
  mutate(Experiment = "STE3 and STE4") %>%
  group_by(Experiment) %>%
  dplyr::summarise(mean_edit = mean(Edit), mean_protein_loss = mean(Protein_Loss),
                   sd_edit = sd(Edit), sd_protein_loss = sd(Protein_Loss),
                   min_edit = min(Edit), max_edit = max(Edit),
                   # median_edit = median(Edit), median_protein_loss = median(Protein_Loss),
                   min_protein_loss = min(Protein_Loss), max_protein_loss = max(Protein_Loss)) %>%
  ungroup() %>%
  dplyr::select(-Experiment) %>%
  mutate_all(~signif(.,digits= 3) %>% scales::percent()) %>%
  mutate(Experiment = "STE3 and STE4") %>%
  dplyr::select(Experiment, everything()) %>%
  datatable()
```

### Figure 3B

Base editing efficiencies or protein loss efficiencies grouped by enzyme and target motif.

```
set.seed(1)
experimental_data %>%
  filter(Experiment != "STE2") %>%
  ggplot(aes(x = Motif, y = Edit, fill = Enzyme)) +
  geom_boxplot(outlier.alpha  = 0) +
  geom_point(pch = 21,position = position_jitter(0.1), alpha= 0.7, fill = "darkgrey", size = 3) +
  xlab("") +
  ylab("Editing of target base") +
  scale_fill_manual(values = c("BE4" = "#1f78b4", "ABE7.10" = "#b2df8a")) +
  scale_y_continuous(limits = c(-0.01, 1.2), labels = scales::percent_format(), breaks = seq(0,1,0.2)) +
  facet_grid(cols = vars(Enzyme), scales = 'free_x', space = "free") +
  theme_bw(base_size = 24) +
  theme(panel.grid.major = element_blank(), panel.grid.minor = element_blank())
```

### Figure 3C

Consistency of editing efficiency and protein loss across all approaches employed here.

```
set.seed(1)
experimental_data %>%
  filter(Experiment != "STE2") %>%
  ggplot(aes(x = Motif, y = Protein_Loss, fill = Enzyme)) +
  geom_boxplot(outlier.alpha  = 0) +
  geom_point(pch = 21,position = position_jitter(0.1), alpha= 0.7, fill = "darkgrey", size = 3) +
  xlab("") +
  ylab("Loss in surface expression") +
  scale_fill_manual(values = c("BE4" = "#1f78b4", "ABE7.10" = "#b2df8a")) +
  scale_y_continuous(limits = c(-0.01, 1.2), labels = scales::percent_format(), breaks = seq(0,1,0.2)) +
  facet_grid(cols = vars(Enzyme), scales = 'free_x', space = "free") +
  theme_bw(base_size = 24) +
  theme(panel.grid.major = element_blank(), panel.grid.minor = element_blank())
```

### Figure 3D

Consistency of editing efficiency and protein loss across all approaches employed here

```
motif_cor_data = experimental_data %>%
  group_by(Enzyme_Motif) %>%
  dplyr::summarize(cor = signif(correlation(Edit, Protein_Loss), 3),
                   pvalue = signif(correlation_pvalue(Edit, Protein_Loss), 3)) %>%
  ungroup() %>%
  mutate(var = Enzyme_Motif) %>%
  mutate(x  = 0.25, y = 0.75) %>%
  mutate(cor = paste0("r = ", cor)) %>%
  mutate(pvalue = paste0("p-value = ", pvalue))

experimental_data %>%
  ggplot(aes(x  = Edit, y = Protein_Loss, fill = Enzyme_Motif)) +
  geom_abline(slope = 1, intercept = 0, linetype = "dashed") +
  geom_smooth(method = "lm", color = "black", aes(fill = NULL)) +
  geom_point(pch = 21) +
  scale_y_continuous(labels = scales::percent_format(), limits = c(0, 1)) +
  scale_x_continuous(labels = scales::percent_format(), limits = c(0, 1)) +
  theme_bw(base_size = 18) +
  geom_label(data = motif_cor_data, aes(x = x, y = y, label = cor, fill = NULL)) +
  xlab("Genetic Editing") +
  ylab("Protein Loss") +
  facet_wrap(.~Enzyme_Motif) +
  theme(aspect.ratio = 1,
        axis.text.x = element_text(hjust = 1, angle = 45),
        legend.position = "none",
        panel.grid.major = element_blank(),
        panel.grid.minor = element_blank()
        )
```

### Figure 3 statistics

Statistics used to make all comparisons in figure 3C

```
### FIGURE 3 STATISTICS ###
### CBE vs. ABE
# equal variance
experimental_data %>% filter(Experiment != "STE2") %>% filter(Motif != "pmSTOP") %>% var.test(Edit ~ Enzyme, data =  .)
```

```
## 
##  F test to compare two variances
## 
## data:  Edit by Enzyme
## F = 1.0253, num df = 39, denom df = 37, p-value = 0.9414
## alternative hypothesis: true ratio of variances is not equal to 1
## 95 percent confidence interval:
##  0.5360215 1.9502472
## sample estimates:
## ratio of variances 
##            1.02527
```

```
experimental_data %>% filter(Experiment != "STE2") %>% filter(Motif != "pmSTOP") %>% t.test(Edit ~ Enzyme, data =  ., var.equal = T)
```

```
## 
##  Two Sample t-test
## 
## data:  Edit by Enzyme
## t = 2.8683, df = 76, p-value = 0.005339
## alternative hypothesis: true difference in means is not equal to 0
## 95 percent confidence interval:
##  0.06086064 0.33740252
## sample estimates:
##     mean in group BE4 mean in group ABE7.10 
##             0.4915000             0.2923684
```

```
experimental_data %>% filter(Experiment != "STE2") %>% filter(Motif != "pmSTOP") %>% var.test(Protein_Loss ~ Enzyme, data =  .)
```

```
## 
##  F test to compare two variances
## 
## data:  Protein_Loss by Enzyme
## F = 1.8205, num df = 39, denom df = 37, p-value = 0.06984
## alternative hypothesis: true ratio of variances is not equal to 1
## 95 percent confidence interval:
##  0.9517687 3.4628915
## sample estimates:
## ratio of variances 
##           1.820487
```

```
experimental_data %>% filter(Experiment != "STE2") %>% filter(Motif != "pmSTOP") %>% t.test(Protein_Loss ~ Enzyme, data =  ., var.equal = T)
```

```
## 
##  Two Sample t-test
## 
## data:  Protein_Loss by Enzyme
## t = 3.7515, df = 76, p-value = 0.0003414
## alternative hypothesis: true difference in means is not equal to 0
## 95 percent confidence interval:
##  0.1254955 0.4095583
## sample estimates:
##     mean in group BE4 mean in group ABE7.10 
##             0.4076680             0.1401411
```

```
### SD vs.SA
## Across ABE and CBE
# Equal variance
# No significant difference in editing between SDs and SAs across ABEs and CBEs
experimental_data %<>% mutate(Motif = as.character(levels(Motif)[Motif]))

experimental_data %>% filter(Experiment != "STE2") %>% filter(Motif != "pmSTOP") %>% var.test(Edit ~ Motif, data =  .)
```

```
## 
##  F test to compare two variances
## 
## data:  Edit by Motif
## F = 1.0712, num df = 33, denom df = 43, p-value = 0.8231
## alternative hypothesis: true ratio of variances is not equal to 1
## 95 percent confidence interval:
##  0.5663223 2.0842249
## sample estimates:
## ratio of variances 
##           1.071242
```

```
# One the cusp of being significant
experimental_data %>% filter(Experiment != "STE2") %>% filter(Motif != "pmSTOP") %>% t.test(Edit ~ Motif, data =  ., var.equal = T)
```

```
## 
##  Two Sample t-test
## 
## data:  Edit by Motif
## t = -0.9703, df = 76, p-value = 0.335
## alternative hypothesis: true difference in means is not equal to 0
## 95 percent confidence interval:
##  -0.21686811  0.07478254
## sample estimates:
## mean in group SA mean in group SD 
##        0.3544118        0.4254545
```

```
### CBE
### Statistics
CBE_editing_mean = experimental_data %>% filter(Enzyme == "BE4") %>% .$Edit %>% mean(., na.rm = T)
CBE_editing_sd = experimental_data %>% filter(Enzyme == "BE4") %>% .$Edit %>% sd(., na.rm = T)

CBE_protein_mean = experimental_data %>% filter(Enzyme == "BE4") %>% .$Protein_Loss %>% mean(., na.rm = T)
CBE_protein_sd = experimental_data %>% filter(Enzyme == "BE4") %>% .$Protein_Loss %>% sd(., na.rm = T)

# Equal variance of data
# Average editing between SD and SA among CBE is n.s.
experimental_data %>% filter(Motif != "pmSTOP" & Enzyme == "BE4") %>% var.test(Edit ~ Motif, data =  .)
```

```
## 
##  F test to compare two variances
## 
## data:  Edit by Motif
## F = 1.1834, num df = 19, denom df = 19, p-value = 0.7174
## alternative hypothesis: true ratio of variances is not equal to 1
## 95 percent confidence interval:
##  0.4683864 2.9896889
## sample estimates:
## ratio of variances 
##           1.183355
```

```
experimental_data %>% filter(Motif != "pmSTOP" & Enzyme == "BE4") %>% t.test(Edit ~ Motif, data =  ., var.equal = T)
```

```
## 
##  Two Sample t-test
## 
## data:  Edit by Motif
## t = 0.41603, df = 38, p-value = 0.6797
## alternative hypothesis: true difference in means is not equal to 0
## 95 percent confidence interval:
##  -0.1585068  0.2405068
## sample estimates:
## mean in group SA mean in group SD 
##            0.512            0.471
```

```
# Equal variance of data

# Average protein loss between SD and SA among CBE is n.s.
experimental_data %>% filter(Motif != "pmSTOP" & Enzyme == "BE4") %>% var.test(Protein_Loss ~ Motif, data =  .)
```

```
## 
##  F test to compare two variances
## 
## data:  Protein_Loss by Motif
## F = 0.81088, num df = 19, denom df = 19, p-value = 0.6523
## alternative hypothesis: true ratio of variances is not equal to 1
## 95 percent confidence interval:
##  0.3209545 2.0486381
## sample estimates:
## ratio of variances 
##          0.8108759
```

```
experimental_data %>% filter(Motif != "pmSTOP" & Enzyme == "BE4") %>% t.test(Protein_Loss ~ Motif, data =  ., var.equal = T)
```

```
## 
##  Two Sample t-test
## 
## data:  Protein_Loss by Motif
## t = -1.2656, df = 38, p-value = 0.2134
## alternative hypothesis: true difference in means is not equal to 0
## 95 percent confidence interval:
##  -0.36787552  0.08484688
## sample estimates:
## mean in group SA mean in group SD 
##        0.3369108        0.4784252
```

```
### ABE
### Statistics
ABE_editing_mean = experimental_data %>% filter(Enzyme == "ABE7.10") %>% .$Edit %>% mean(., na.rm = T)
ABE_editing_sd = experimental_data %>% filter(Enzyme == "ABE7.10") %>% .$Edit %>% sd(., na.rm = T)

ABE_protein_mean = experimental_data %>% filter(Enzyme == "ABE7.10") %>% .$Protein_Loss %>% mean(., na.rm = T)
ABE_protein_sd = experimental_data %>% filter(Enzyme == "ABE7.10") %>% .$Protein_Loss %>% sd(., na.rm = T)

experimental_data %>%
  filter(Enzyme == "BE4" & Motif == "pmSTOP") %$%
  cor.test(x = Edit, y = Protein_Loss)
```

```
## 
##  Pearson's product-moment correlation
## 
## data:  Edit and Protein_Loss
## t = 2.3501, df = 34, p-value = 0.02471
## alternative hypothesis: true correlation is not equal to 0
## 95 percent confidence interval:
##  0.05162958 0.62553526
## sample estimates:
##       cor 
## 0.3738244
```

```
# Data does not have equal variance
# Average editing between SD and SA among ABE is significantly different
experimental_data %>% filter(Motif != "pmSTOP" & Enzyme == "ABE7.10") %>% var.test(Edit ~ Motif, data =  .)
```

```
## 
##  F test to compare two variances
## 
## data:  Edit by Motif
## F = 0.22385, num df = 13, denom df = 23, p-value = 0.007413
## alternative hypothesis: true ratio of variances is not equal to 1
## 95 percent confidence interval:
##  0.08844827 0.65030507
## sample estimates:
## ratio of variances 
##          0.2238452
```

```
experimental_data %>% filter(Motif != "pmSTOP" & Enzyme == "ABE7.10") %>% t.test(Edit ~ Motif, data =  ., var.equal = F)
```

```
## 
##  Welch Two Sample t-test
## 
## data:  Edit by Motif
## t = -3.2475, df = 34.937, p-value = 0.002573
## alternative hypothesis: true difference in means is not equal to 0
## 95 percent confidence interval:
##  -0.41964306 -0.09678551
## sample estimates:
## mean in group SA mean in group SD 
##        0.1292857        0.3875000
```

```
# Data does not have equal variance
# Average protein loss between SD and SA among ABE is significantly different
experimental_data %>% filter(Motif != "pmSTOP" & Enzyme == "ABE7.10") %>% var.test(Protein_Loss ~ Motif, data =  .)
```

```
## 
##  F test to compare two variances
## 
## data:  Protein_Loss by Motif
## F = 0.011774, num df = 13, denom df = 23, p-value = 2.041e-10
## alternative hypothesis: true ratio of variances is not equal to 1
## 95 percent confidence interval:
##  0.004652251 0.034205107
## sample estimates:
## ratio of variances 
##         0.01177394
```

```
experimental_data %>% filter(Motif != "pmSTOP" & Enzyme == "ABE7.10") %>% t.test(Protein_Loss ~ Motif, data =  ., var.equal = F)
```

```
## 
##  Welch Two Sample t-test
## 
## data:  Protein_Loss by Motif
## t = -2.8099, df = 23.921, p-value = 0.009722
## alternative hypothesis: true difference in means is not equal to 0
## 95 percent confidence interval:
##  -0.31584793 -0.04831611
## sample estimates:
## mean in group SA mean in group SD 
##       0.02514189       0.20722392
```

```
### SD vs. SA vs. pmSTOP
# Just focus on CBE for fair comparison

# Equal variance of data
# Average editing between SD and pmSTOP is n.s.
experimental_data %>% filter(Motif != "SA" & Enzyme == "BE4") %>% var.test(Edit ~ Motif, data =  .)
```

```
## 
##  F test to compare two variances
## 
## data:  Edit by Motif
## F = 1.0904, num df = 35, denom df = 19, p-value = 0.8637
## alternative hypothesis: true ratio of variances is not equal to 1
## 95 percent confidence interval:
##  0.4621588 2.3336483
## sample estimates:
## ratio of variances 
##           1.090361
```

```
experimental_data %>% filter(Motif != "SA" & Enzyme == "BE4") %>% t.test(Edit ~ Motif, data =  ., var.equal = T)
```

```
## 
##  Two Sample t-test
## 
## data:  Edit by Motif
## t = -0.8458, df = 54, p-value = 0.4014
## alternative hypothesis: true difference in means is not equal to 0
## 95 percent confidence interval:
##  -0.24397837  0.09920059
## sample estimates:
## mean in group pmSTOP     mean in group SD 
##            0.3986111            0.4710000
```

```
# Variance of data is equal
# Average protein loss between SD and pmSTOP among CBE is significant
experimental_data %>% filter(Motif != "SA" & Enzyme == "BE4") %>% var.test(Protein_Loss ~ Motif, data =  .)
```

```
## 
##  F test to compare two variances
## 
## data:  Protein_Loss by Motif
## F = 0.50361, num df = 35, denom df = 19, p-value = 0.07704
## alternative hypothesis: true ratio of variances is not equal to 1
## 95 percent confidence interval:
##  0.2134575 1.0778431
## sample estimates:
## ratio of variances 
##          0.5036057
```

```
experimental_data %>% filter(Motif != "SA" & Enzyme == "BE4") %>% t.test(Protein_Loss ~ Motif, data =  ., var.equal = T)
```

```
## 
##  Two Sample t-test
## 
## data:  Protein_Loss by Motif
## t = -3.3294, df = 54, p-value = 0.001573
## alternative hypothesis: true difference in means is not equal to 0
## 95 percent confidence interval:
##  -0.4552842 -0.1130507
## sample estimates:
## mean in group pmSTOP     mean in group SD 
##            0.1942577            0.4784252
```

```
# Just focus on CBE for fair comparison
# Equal variance of data
# Average editing between SA and pmSTOP is n.s.
# Although close
experimental_data %>% filter(Motif != "SD" & Enzyme == "BE4") %>% var.test(Edit ~ Motif, data =  .)
```

```
## 
##  F test to compare two variances
## 
## data:  Edit by Motif
## F = 0.92142, num df = 35, denom df = 19, p-value = 0.8088
## alternative hypothesis: true ratio of variances is not equal to 1
## 95 percent confidence interval:
##  0.3905495 1.9720607
## sample estimates:
## ratio of variances 
##          0.9214152
```

```
experimental_data %>% filter(Motif != "SD" & Enzyme == "BE4") %>% t.test(Edit ~ Motif, data =  ., var.equal = T)
```

```
## 
##  Two Sample t-test
## 
## data:  Edit by Motif
## t = -1.2862, df = 54, p-value = 0.2038
## alternative hypothesis: true difference in means is not equal to 0
## 95 percent confidence interval:
##  -0.29012976  0.06335198
## sample estimates:
## mean in group pmSTOP     mean in group SA 
##            0.3986111            0.5120000
```

```
# Variance of data is equal, although close
# Average protein loss between SA and pmSTOP among CBE is n.s. significant but close
experimental_data %>% filter(Motif != "SD" & Enzyme == "BE4") %>% var.test(Protein_Loss ~ Motif, data =  .)
```

```
## 
##  F test to compare two variances
## 
## data:  Protein_Loss by Motif
## F = 0.62106, num df = 35, denom df = 19, p-value = 0.2175
## alternative hypothesis: true ratio of variances is not equal to 1
## 95 percent confidence interval:
##  0.2632431 1.3292331
## sample estimates:
## ratio of variances 
##          0.6210638
```

```
experimental_data %>% filter(Motif != "SD" & Enzyme == "BE4") %>% t.test(Protein_Loss ~ Motif, data =  ., var.equal = T)
```

```
## 
##  Two Sample t-test
## 
## data:  Protein_Loss by Motif
## t = -1.7599, df = 54, p-value = 0.08408
## alternative hypothesis: true difference in means is not equal to 0
## 95 percent confidence interval:
##  -0.30515922  0.01985296
## sample estimates:
## mean in group pmSTOP     mean in group SA 
##            0.1942577            0.3369108
```

## Figure 4

### Legend

Figure 4. Context dependencies of base editors and BE-splice target motifs. (a) Dinucleotide context dependencies of rAPOBEC1-BE4. Dinucleotide context is defined by the identity of the target base, and the identity of the base immediately preceding the target. Results normalized and aggregated across published results and our own work. Smoothed distributions generated using LOESS regression with span = 0.5. (b) Preceding dinucleotide context dependencies of TadAWT-TadAEvo-ABE7.10. Results normalized and aggregated across published results and our own work. Smoothed distributions generated using LOESS regression with span = 0.45. N = 6 papers, 102 guides, and 447 edits in the analysis. (c) Logo plots of the pentanucleotide motif for each enzyme and target motif combination in this work. The pentanucleotide motif is oriented with respect to the sgRNA protospacer, independent of how a protospacer is oriented with respect to the direction of gene transcription. The heights of bases are proportional to the prevalence of the base at that position in the target site. (d) Boxplot of BE-Hive predicted editing efficiencies between BE4 and ABE7.10 gRNAs. Boxplot center lines represent the median, box limits represent the upper and lower quartiles, and whiskers define the 1.5x interquartile range. Analyzed by two-sided Wilcoxon rank sum test due to the non-normal distribution of data. N = 275 BE4 edits, and 342 ABE7.10 edits. (e) Comparisons between observed values in meta-analysis and BE-Hive or Honeycomb predicted scorings. Data plotted on a logit scale to better observe relationship of data. Spearman’s rank correlation coefficient (ρ) is shown. Trend line is linear model line of best fit, grey shading is 95% CI of the mean. Source data and code are available in the Source Data file.

### Figure 4A

Preceding dinucleotide context dependencies of rAPOBEC1-BE4

```
# CBE base editing by position, by dinucleotide
# Need to break up in the actual figure
cbe_fit = NULL

cbe_data %>%
  flushOutData(., "C") %>%
  filter(!is.na(Dinucleotide)) %>%
  ggplot(aes(x = Position, y = Edit_norm, fill = Dinucleotide)) +
  ylab("Percent Editing") +
  xlab("Position in Protospacer") +
  geom_bar(stat = "summary", fun.y = "mean", color  = "black", alpha = 0.7) +
  stat_smooth(aes(outfit=cbe_fit<<-..y..), span = 0.5, color = "black") + 
  geom_point(alpha = 0.7) +
  scale_y_continuous(limits  = c(-0.01, 1.01), breaks = seq(0,1, 0.2), labels = scales::percent_format()) +
  scale_x_continuous(breaks = seq(0, 20, 2)) +
  coord_cartesian(xlim =  c(1,20), clip = "on")  +
  scale_fill_manual(values = c("All" = "white", "AC" = "#4daf4a", "CC" = "#377eb8", "GC" = "grey", "TC" = "#e41a1c")) +
  labs(fill = "Pre-dinucleotide") +
  theme_bw(base_size = 18) +
  theme(aspect.ratio = 1/2,
        panel.grid.major = element_blank(), panel.grid.minor = element_blank()) +
  facet_grid(cols = vars(Dinucleotide))
```

```
## make a dataframe for predicting edits
cbe_prediction = data.frame(predicted_editing = cbe_fit) %>%
  mutate(Position = rep(rep(1:20, each = 4), times = 5)) %>%
  mutate(letter = rep(rep(c("a", "b", "c", "d"), times = 20), times = 5)) %>%
  mutate(Dinucleotide = rep(c("All", "TC", "CC", "AC", "GC"), each = 80)) %>%
  filter(letter == "d") %>%
  dplyr::select(-letter) %>%
  mutate(predicted_editing = {ifelse(predicted_editing < 0, 0, predicted_editing)}) %>%
  mutate(Enzyme = "BE4")
```

### Figure 4B

Preceding dinucleotide context dependencies of TadAWT-TadA-Evo-ABE7.10

```
# ABE base editing by position, by dinucleotide
abe_fit = NULL

abe_data %>%
  flushOutData(., "A") %>%
  filter(!is.na(Dinucleotide)) %>%
  ggplot(aes(x = Position, y = Edit_norm, fill = Dinucleotide)) +
  ylab("Percent Editing") +
  xlab("Position in Protospacer") +
  geom_bar(stat = "summary", fun.y = "mean", color  = "black", alpha = 0.7) +
  stat_smooth(aes(outfit=abe_fit<<-..y..), span = 0.4, color = "black") + 
  geom_point() +
  scale_y_continuous(limits  = c(-0.01, 1.01), breaks = seq(0,1,0.2), labels = scales::percent_format()) +
  scale_x_continuous(breaks = seq(0, 20, 2)) +
  coord_cartesian(xlim =  c(1,20), clip = "on")  +
  scale_fill_manual(values = c("All" = "white", "AA" = "#4daf4a", "CA" = "#377eb8", "GA" = "grey", "TA" = "#e41a1c")) +
  labs(fill = "Pre-dinucleotide") +
  theme_bw(base_size = 18) +
  theme(aspect.ratio = 1/2,
        panel.grid.major = element_blank(), panel.grid.minor = element_blank()) +
  facet_grid(cols = vars(Dinucleotide))
```

```
## make a dataframe for predicting edits
abe_prediction = data.frame(predicted_editing = abe_fit) %>%
  mutate(Position = rep(rep(1:20, each = 4), times = 5)) %>%
  mutate(letter = rep(rep(c("a", "b", "c", "d"), times = 20), times = 5)) %>%
  mutate(Dinucleotide = rep(c("All", "TA", "CA", "GA", "AA"), each = 80)) %>%
  filter(letter == "d") %>%
  dplyr::select(-letter) %>%
  mutate(predicted_editing = {ifelse(predicted_editing < 0, 0, predicted_editing)}) %>%
  mutate(Enzyme = "ABE7.10")
```

### Figure 4C

Logo plots of the pentanucleotide motif for each enzyme and target motif combination in this work.

BE4 SD Motif

```
# figure_4Ci
plotLogo(experimental_data, 1)
```

BE4 SA Motif

```
# figure_4Cii
plotLogo(experimental_data, 2)
```

BE4 pmSTOP Motif

```
# figure_4Ciii
plotLogo(experimental_data, 3)
```

ABE7.10 SD Motif

```
# figure_4Civ
plotLogo(experimental_data, 4)
```

ABE7.10 SA Motif

```
# figure_4Cv
plotLogo(experimental_data, 5)
```

### Figure 4 statistics

```
n_guides = bind_rows(abe_data, cbe_data) %>%
  .$Protospacer %>%
  unique() %>%
  length

n_papers = bind_rows(abe_data, cbe_data) %>%
  .$Paper %>%
  unique() %>%
  length %>%
  {. + 1}

n_edits = bind_rows(abe_data, cbe_data) %>%
  dplyr::select(Protospacer, Position) %>%
  distinct %>%
  nrow()

n_guides
```

```
## [1] 102
```

```
n_papers
```

```
## [1] 1
```

```
n_edits
```

```
## [1] 447
```

### Figure 4D

Boxplot of BE-Hive predicted editing efficiencies between BE4 and ABE7.10 gRNAs

```
# BE4 analysis
## Read and manipulate data

# Establish the guides that were unique to this work
BE4_new_guides = BE4_guides %>% filter(Paper == "this work") %>% .$Protospacer

### clean data
BE4_flushedOut = BE4_guides %>%
  # convert to upper case 
  mutate(Protospacer = toupper(Protospacer)) %>%
  # Establish the edited base as T
  mutate(Edit = `T`) %>%
  
  # Establish the maximum edit for each cell type in each paper
  group_by(`Cell type`, Paper) %>%
  mutate(paper_max = max(Edit)) %>%
  ungroup %>%
  
  # Normalize the edits to the max edit observed in the paper
  mutate(Edit_norm = Edit/paper_max) %>%
  
  # For each position in each unique guide, calculate the average normalized edit
  # This averages across replicates
  group_by(Position, Protospacer) %>%
  dplyr::summarise(Edit_norm = mean(Edit_norm)) %>%
  ungroup() %>%
  arrange(Protospacer) %>%
  
  #Flush out the data
  flushOutData(., "C") %>%
  filter(!is.na(Dinucleotide)) %>%
  filter(Dinucleotide != "All") %>%
  distinct() %>%
  mutate(source = {ifelse(Protospacer %in% BE4_new_guides, "this work", "et al.")})

# merge data
BE4_guides_2 = BE4_flushedOut %>%
  inner_join(., BE4_guides_predicted)

BE4_guides_summarized = lapply(FUN = pullPredictions, data = BE4_guides_2, X = 1:nrow(BE4_guides_2), base = "C") %>%
  plyr::ldply(., "data.frame") %>%
  tibble()


### ABE data
ABE_new_guides = ABE_guides %>% filter(Paper == "this work") %>% .$Protospacer

### clean data
ABE_flushedOut = ABE_guides %>%
  mutate(Protospacer = toupper(Protospacer)) %>%
  mutate(Edit = `G`) %>%
  
  # Establish the maximum edit for each cell type in each paper
  group_by(`Cell type`, Paper) %>%
  mutate(paper_max = max(Edit)) %>%
  ungroup %>%
  
  # Normalize the edits to the max edit observed in the paper
  mutate(Edit_norm = Edit/paper_max) %>%
  
  # For each position in each unique guide, calculate the average normalized edit
  group_by(Position, Protospacer) %>%
  dplyr::summarise(Edit_norm = mean(Edit_norm)) %>%
  ungroup() %>%
  arrange(Protospacer) %>%
  
  #Flush out the data
  flushOutData(., "A") %>%
  filter(!is.na(Dinucleotide)) %>%
  filter(Dinucleotide != "All") %>%
  distinct() %>%
  mutate(source = {ifelse(Protospacer %in% ABE_new_guides, "this work", "et al.")})

# merge data
ABE_guides_2 = ABE_flushedOut %>%
  inner_join(., ABE_guides_predicted)

ABE_guides_summarized = lapply(FUN = pullPredictions, data = ABE_guides_2, X = 1:nrow(ABE_guides_2), base = "A") %>%
  plyr::ldply(., "data.frame") %>%
  tibble()

logistic_adjust = -2

meta_analysis = ABE_guides_summarized %>%
  mutate(Edit_predicted = pG) %>%
  bind_rows(., BE4_guides_summarized %>%
              mutate(Edit_predicted = pT)
  ) %>%
  mutate(enzyme = {ifelse(enzyme == "ABE", "ABE7.10", "BE4")}) %>%
  dplyr::select(Protospacer, Position,  Edit_norm, Edit_predicted, enzyme) %>%
  mutate(motif = substr(Protospacer, start = Position - 3, stop = Position + 3)) %>%
  filter(nchar(motif) == 7) %>%
  inner_join(., motif_weights %>%
               dplyr::select(motif, motif_weight)
  ) %>%
  inner_join(., position_weights %>%
               dplyr::rename(Position = position)
  ) %>%
  mutate(Honeycomb = probability(motif_weight + position_weight + logistic_adjust)) %>%
  mutate(dataset = "Meta-analysis") %>%
  dplyr::rename(Observed = Edit_norm, `BE-Hive` = Edit_predicted, Enzyme = enzyme) %>%
  dplyr::select(Enzyme, Observed, `BE-Hive`, Honeycomb, dataset)

# Compare the predicted values for ABE and BE4
figure_4D_data = bind_rows(
  ABE_guides_summarized %>%
    dplyr::select(Position, pG, Dinucleotide, Protospacer) %>%
    mutate(enzyme = "ABE7.10") %>%
    dplyr::rename(Edit = pG),
  BE4_guides_summarized %>%
    dplyr::select(Position, pT, enzyme, Dinucleotide, Protospacer) %>%
    dplyr::rename(Edit = pT)
) %>%
  mutate(enzyme = factor(enzyme, levels = c("BE4", "ABE7.10")))

figure_4D_pvalue = figure_4D_data %$%
  wilcox.test(Edit~enzyme) %>%
  .$p.value
```

```
figure_4D_data %>%
  ggplot(aes(x = enzyme, y = Edit, fill = enzyme)) +
  geom_boxplot(fill = "white") +
  geom_quasirandom(pch = 21, alpha = 0.7) +
  scale_fill_brewer(palette = "Set1") +
  scale_y_continuous(limits = c(0,1.2), breaks = seq(0,1,0.2), labels = scales::percent_format()) +
  ylab("Predicted Efficiency (BE-hive)") +
  xlab("Enzyme") +
  theme_bw(base_size = 24) +
  theme(aspect.ratio = 2, panel.grid = element_blank())
```

Number of edits by each enzyme

```
figure_4D_data %>%
  dplyr::select(enzyme, Position, Protospacer) %>%
  distinct() %>%
  group_by(enzyme) %>%
  dplyr::summarise(number_of_edits = length(enzyme)) %>%
  datatable()
```

```
n_be4_edits = sum(figure_4D_data$enzyme == "BE4")
```

### Figure 4E

Comparisons between observed values in meta-analysis and BE-Hive or Honeycomb predicted scorings

```
plotFigure4E("Observed", "BE-Hive")
```

```
plotFigure4E("Observed", "Honeycomb")
```

```
plotFigure4E("Honeycomb", "BE-Hive")
```

## Figure 5

### Legend

Figure 5. Consistency of editing efficiency and protein loss across mRNA and protein regions. (a) Scatter plots of protein loss and editing efficiency by exon grouping across all base editor approaches employed. The strongest relationship is observed among middle exons, while the weakest is observed in the last exon. Pearson’s correlation coefficient (r) is shown. Error bands represent 95% CI of the mean. (b) Error in protein loss as a function of editing efficiency across each exon group. Boxplot center lines represent the median, box limits represent the upper and lower quartiles, and whiskers define the 1.5x interquartile range. The least error is observed among middle exons, while the greatest is observed in the last exon. N = 57 unique enzyme-guide combinations with 2 independent donors. (c) Scatter plots of the protein loss and editing efficiency grouped by where each BE sgRNA maps to the TCR-CD3 and MHC Class I structures. sgRNAs that map to transmembrane and extracellular regions exhibit the greatest consistency between protein loss and base editing efficiency. Pearson’s correlation coefficient (r) is shown. Error bands represent 95% CI of the mean. Source data and code are available in the Source Data file.

### Figure 5A

Scatter plots of protein loss and editing efficiency by exon group across all base editor approaches employed.

```
### Figure 5A
# Position dependent effects of correlation of base editing

position_cor_data = experimental_data %>%
  group_by(Exon_Class) %>%
  dplyr::summarize(cor = signif(correlation(Edit, Protein_Loss), 3),
                   pvalue = signif(correlation_pvalue(Edit, Protein_Loss), 3),
                   n = length(Edit)) %>%
  mutate(cor = paste0("r = ", cor)) %>%
  mutate(pvalue = paste0("p-value = ", pvalue))

experimental_data %>%
  filter(Experiment != "STE2") %>%
  ggplot(aes(x  = Edit, y = Protein_Loss, fill = Exon_Class)) +
  geom_abline(slope = 1, intercept = 0, linetype = "dashed") +
  geom_smooth(method = "lm", color = "black", aes(fill = NULL)) +
  geom_point(pch = 21) +
  scale_y_continuous(labels = scales::percent_format(), limits = c(0, 1)) +
  scale_x_continuous(labels = scales::percent_format(), limits = c(0, 1)) +
  theme_bw(base_size = 12) +
  geom_label(data = position_cor_data, aes(label = cor, fill = NULL), x = 0.25, y = 0.75) +
  xlab("Genetic Editing") +
  ylab("Protein Loss") +
  facet_grid(cols = vars(Exon_Class)) +
  theme(aspect.ratio = 1,
        axis.text.x = element_text(hjust = 1, angle = 45),
        legend.position = "none",
        panel.grid.major = element_blank(),
        panel.grid.minor = element_blank()
  )
```

### Figure 5B

Error in protein loss as a function of editing efficiency across each exon group

```
### Figure 5B
experimental_data %>%
  filter(Experiment != "STE2") %>%
  mutate(diff = abs(Protein_Loss - Edit)) %>%
  ggplot(aes(x = Exon_Class, y = diff, fill = Exon_Class)) +
  geom_boxplot(outlier.alpha = 0, alpha = 0.7) +
  geom_line(aes(x = as.numeric(Exon_Class), y = diff, fill = NULL), fun.y = "median", stat = "summary", lwd = 1.5) +
  geom_point() +
  ylab("Error (|Protein Loss - Editing|)") +
  xlab("Exon") +
  scale_y_continuous(limits = c(0,1), breaks = seq(0,1, 0.2), labels = scales::percent_format()) +
  theme_classic(base_size = 24) +
  theme(legend.position = "none")
```

```
exon_group.lm = experimental_data %>%
  filter(Experiment != "STE2") %>%
  mutate(diff = abs(Protein_Loss - Edit)) %>%
  lm(diff ~ 0 + Exon_Group, data = .)

exon_group.lm %>%
  summary() %>%
  .[[4]] %>%
  as.data.frame() %>%
  rownames_to_column("Exon_Group") %>%
  mutate(Exon_Group = gsub("Exon_Group", "", Exon_Group)) %>%
  as_tibble %>%
  datatable()
```

### Figure 5C

Scatter plots of the protein loss and editing efficiency grouped by where each BE sgRNA maps to the TCR-CD3 and MHC Class I structures

```
### Figure 5C
domain_cor_data = experimental_data %>%
  filter(Experiment != "STE2") %>%
  filter(Domain != "UTR") %>%
  group_by(Domain) %>%
  dplyr::summarize(cor = signif(correlation(Edit, Protein_Loss), 3),
                   pvalue = signif(correlation_pvalue(Edit, Protein_Loss), 3)) %>%
  mutate(cor = paste0("r = ", cor)) %>%
  mutate(pvalue = paste0("p-value = ", pvalue))

experimental_data %>%
  filter(Experiment != "STE2") %>%
  filter(Domain != "UTR") %>%
  mutate(Domain = factor(Domain, levels = c("Extracellular", "Transmembrane", "Intracellular"))) %>%
  ggplot(aes(x  = Edit, y = Protein_Loss, fill = Domain)) +
  geom_abline(slope = 1, intercept = 0, linetype = "dashed") +
  geom_smooth(method = "lm", color = "black", aes(fill = NULL)) +
  geom_point(pch = 21, size = 2) +
  scale_y_continuous(labels = scales::percent_format(), limits = c(0, 1)) +
  scale_x_continuous(labels = scales::percent_format(), limits = c(0, 1)) +
  theme_bw(base_size = 18) +
  geom_label(data = domain_cor_data, aes(label = cor, fill = NULL), x = 0.25, y = 0.75) +
  xlab("Genetic Editing") +
  ylab("Protein Loss") +
  facet_grid(.~Domain) +
  theme(aspect.ratio = 1,
        axis.text.x = element_text(hjust = 1, angle = 45),
        legend.position = "none",
        panel.grid.major = element_blank(),
        panel.grid.minor = element_blank()
  )
```

## Figure 6

### Legend

Figure 6. Using ABEs and CBEs to disrupt the intracellular protein CISH in the K562 cell line. (a) Diagram of CISH immunohibitory pathway73. (b) Mapping of gRNAs to CISH locus. (c) Editing efficiencies with gRNAs paired with ABE7.10, ABE8e, and BE4. AAVS1 is an inert locus control. (d) Taqman expression assays of CISH exon boundaries. Data normalized to AAVS1 control. N = 2 biological replicates, each with 3 technical replicates. (e) Representative gel image of whole cDNA amplification of edited samples with altered isoforms from two independent biological replicates. See Supplementary Fig.15 for uncropped gel image. (f) Relative protein expression quantified from digital western blot of CISH. CISH expression normalized within-samples to β-ACTIN and between-samples to AAVS1 control. Height of bars represents mean of N = 2 biological replicates. (g) Representative figital western blot of CISH and β-ACTIN from two independent biological replicates. See Supplementary Fig.17 for uncropped western blot images. Source data and code are available in the Source Data file.

### Figure 6A

Diagram of CISH immunoinhibitory pathway

### Figure 6B

Mapping of gRNAs to CISH locus

### Figure 6C

Editing efficiencies with gRNAs paired with ABE7.10, ABE8e, and BE4

```
CISH %>%
  ggplot(aes(x = Guide, y = Edit, fill = Enzyme)) +
  geom_bar(stat = "summary", fun.y = "mean", position = "dodge", color = "black") +
  scale_fill_brewer(palette = 1) +
  geom_point(position = position_dodge2(0.2), color = "black", size = 3) +
  facet_grid(cols = vars(Enzyme), space = "fixed", scale = "free") + 
  scale_y_continuous(breaks = seq(0,1,0.2), labels = scales::percent_format()) +
  ylab("Editing Efficiency") +
  xlab("")+
  theme_bw(base_size = 28) +
  theme(axis.text.x = element_text(hjust = 1, angle = 45),
        legend.position = "none",
        panel.grid = element_blank(),
        aspect.ratio = 0.9)
```

### Figure 6D

Taqman expression assays of CISH exon boundaries

```
taqman2 = CISH_taqman %>%
  filter(Probe == "B-ACTIN") %>%
  dplyr::select(Sample, ave_Cq) %>%
  dplyr::rename(bactin_ave_Cq = ave_Cq)

taqman3 = inner_join(CISH_taqman, taqman2) %>%
  mutate(delta_Cq = ave_Cq - bactin_ave_Cq)

taqman4 = taqman3 %>%
  dplyr::select(Sample, Control, Probe, delta_Cq) %>%
  filter(Sample == Control) %>%
  dplyr::rename(ctrl_delta_Cq = delta_Cq) %>%
  dplyr::select(-Sample) %>%
  inner_join(taqman3, .) %>%
  mutate(delta_delta_Cq = delta_Cq - ctrl_delta_Cq) %>%
  mutate(`2^(-ΔΔCt)` = 2^(-delta_delta_Cq)) %>%
  mutate(`Fold of B-Actin` = 2^(bactin_ave_Cq - ave_Cq)) %>%
  mutate(`Fold of B-Actin` = {ifelse(is.na(`Fold of B-Actin`), 0, `Fold of B-Actin`)}) %>%
  mutate(`2^(-ΔΔCt)` = {ifelse(is.na(`2^(-ΔΔCt)`), 0, `2^(-ΔΔCt)`)})
```

```
taqman4 %>%
  filter(Junction != "β-Actin") %>%
  filter(Junction == "Ex.1a – Ex.2" | Junction == "Ex.2 – Ex.3") %>%
  ggplot(aes(x = Guide, y = `2^(-ΔΔCt)`, fill = Junction)) +
  geom_bar(stat = "summary", fun.y = "mean", position = "dodge", color = "black") +
  scale_fill_brewer(palette = 3) +
  # geom_errorbar( position = position_dodge(), colour="black") +
  geom_point(position = position_dodge(0.9), color = "black", size = 3) +
  geom_hline(yintercept = 1, linetype = "dashed") +
  facet_grid(cols = vars(Enzyme), space = "fixed", scale = "free") + 
  xlab("")+
  ylab("Relative Splice Expression") +
  theme_bw(base_size = 28) +
  theme(axis.text.x = element_text(hjust = 1, angle = 45),
        legend.position = "none", 
        panel.grid = element_blank(),
        aspect.ratio = 0.9)
```

### Figure 6E

Gel image of whole cDNA amplification of edited samples with altered isoforms

### Figure 6F

Relative protein expression quantified from digital western blot of CISH

```
CISH %>%
  gather(Isoform, Expression, `Normalized_CISH-1`:`Normalized_CISH-2`) %>%
  filter(Isoform != 'Normalized_CISH-2') %>%
  ggplot(aes(x = Guide, y = Expression, fill = Isoform)) +
  geom_bar(stat = "summary", fun.y = "mean", position = "dodge", color = "black") +
  scale_fill_brewer(palette = 3) +
  # geom_errorbar( position = position_dodge(), colour="black") +
  geom_point(position = position_dodge(0.9), color = "black", size = 3) +
  facet_grid(cols = vars(Enzyme), space = "fixed", scale = "free") + 
  scale_y_continuous(breaks = seq(0,1.4,0.2), labels = scales::percent_format()) +
  geom_hline(yintercept = 1, linetype = "dashed") +
  ylab("Relative Protein Expression") +
  xlab("")+
  theme_bw(base_size = 28) +
  theme(axis.text.x = element_text(hjust = 1, angle = 45),
        panel.grid = element_blank(),
        legend.position = "none",
        aspect.ratio = 0.9)
```

### Figure 6G

Digital western blot of CISH and β-ACTIN

## Figure 7

### Legend

Figure 7. Disrupting genes with base editors. (a) Conserved genetic elements that can be targeted for gene disruption by base editors. Elements that have been targeted in publications are cited, while additional conserved elements that have not been validated at the time of this publication are indicated as to be determined (TBD). Elements include enhancers74, -35 element, -10 element, start codon75, Splice donor15,24, Branch point24, Pyrimidine tract24, Splice acceptor15,22,24, Nonsense20,21, Missense58, Stop codon, pA signal, pA site.

# Supplementary Figures

## Figure S1

Diagram of Splicer v1.0.0

## Figure S2

Distribution of BE-splice sgRNA density across all genes by BE-splice approach

### Figure S2A

CBE splice donors

```
supplementaryFigure2and3(guides, "BE3", "donor", "S2")
```

### Figure S2B

ABE splice donors

```
supplementaryFigure2and3(guides, "ABE", "donor", "S2")
```

### Figure S2C

CBE splice acceptors

```
supplementaryFigure2and3(guides, "BE3", "acceptor", "S2")
```

### Figure S2D

ABE splice acceptors

```
supplementaryFigure2and3(guides, "ABE", "acceptor", "S2")
```

## Figure S3

### Figure S3A

CBE splice donors

```
supplementaryFigure2and3(guides, "BE3", "donor", "S3")
```

### Figure S3B

ABE splice donors

```
supplementaryFigure2and3(guides, "ABE", "donor", "S3")
```

### Figure S3C

CBE splice acceptors

```
supplementaryFigure2and3(guides, "BE3", "acceptor", "S3")
```

### Figure S3D

ABE splice acceptors

```
supplementaryFigure2and3(guides, "ABE", "acceptor", "S3")
```

## Figure S4

Representative gating strategy for flow cytometry

## Figure S5

Dinucleotide context dependencies of rAPOBEC1-BE4 and TadAWT-TadAEvo-ABE7.10

### Figure S5A

Summary of linear model of rAPOBEC1-BE4 editing efficiency as a function of pre-dinucleotide context

```
cbe_data %>%
  flushOutData(., "C")  %>%
  lm(Edit_norm ~ 0 + Dinucleotide, data = .) %>%
  summary() %>%
  .[[4]] %>%
  as.data.frame() %>%
  mutate(Dinucleotide = c("All", "TC", "CC", "AC", "GC")) %>%
  dplyr::rename(`Average Editing` = Estimate) %>%
  dplyr::select(Dinucleotide, everything()) %>%
  mutate(`Average Editing` = scales::percent(`Average Editing`),
         `Std. Error` = scales::percent(`Std. Error`),
         `t value` = signif(`t value`, 3),
         `Pr(>|t|)` = signif(`Pr(>|t|)`, 3)
  )
```

```
##                 Dinucleotide Average Editing Std. Error t value Pr(>|t|)
## DinucleotideAll          All           17.4%     1.425%   12.20 7.08e-31
## DinucleotideTC            TC           28.7%     3.073%    9.33 1.83e-19
## DinucleotideCC            CC           21.1%     2.676%    7.88 1.43e-14
## DinucleotideAC            AC           13.8%     2.909%    4.73 2.72e-06
## DinucleotideGC            GC            6.9%     3.051%    2.25 2.48e-02
```

### Figure S5B

Summary of linear model of TadAWT-TadAEvo-ABE7.10 editing efficiency as a function of pre-dinucleotide context

```
abe_data %>%
  flushOutData(., "A")  %>%
  lm(Edit_norm ~ 0 + Dinucleotide, data = .) %>%
  summary() %>%
  .[[4]] %>%
  as.data.frame() %>%
  mutate(Dinucleotide = c("All", "TA", "CA", "AA", "GA")) %>%
  dplyr::rename(`Average Editing` = Estimate) %>%
  dplyr::select(Dinucleotide, everything()) %>%
  mutate(`Average Editing` = scales::percent(`Average Editing`),
         `Std. Error` = scales::percent(`Std. Error`),
         `t value` = signif(`t value`, 3),
         `Pr(>|t|)` = signif(`Pr(>|t|)`, 3)
  )
```

```
##                 Dinucleotide Average Editing Std. Error t value Pr(>|t|)
## DinucleotideAll          All          12.56%     1.329%    9.45 4.10e-20
## DinucleotideTA            TA          24.49%     2.927%    8.37 2.87e-16
## DinucleotideCA            CA          13.45%     2.727%    4.93 9.96e-07
## DinucleotideAA            AA          10.19%     2.697%    3.78 1.70e-04
## DinucleotideGA            GA           6.31%     2.469%    2.55 1.08e-02
```

### Figure S5C

Distribution of APOBEC1-BE4 editing efficiency across the protospacer by post-dinucleotide context

```
cbe_data %>%
  flushOutData(., "C") %>%
  filter(!is.na(PostDinucleotide)) %>%
  ggplot(aes(x = Position, y = Edit_norm, fill = PostDinucleotide)) +
  ylab("Percent Editing") +
  xlab("Position in Protospacer") +
  geom_bar(stat = "summary", fun.y = "mean", color  = "black", alpha = 0.7) +
  stat_smooth(aes(outfit=tmp_fit<<-..y..), span = 0.5, color = "black") + 
  geom_point() +
  scale_y_continuous(limits  = c(0, 1), breaks = seq(0,1, 0.2), labels = scales::percent_format()) +
  scale_x_continuous(breaks = seq(0, 20, 2)) +
  coord_cartesian(xlim =  c(1,20), clip = "on")  +
  scale_fill_manual(values = c("All" = "white", "CA" = "#4daf4a", "CC" = "#377eb8", "CG" = "grey", "CT" = "#e41a1c")) +
  labs(fill = "Post-dinucleotide") +
  theme_bw(base_size = 18) +
  theme(aspect.ratio = 1/2,
        panel.grid.major = element_blank(), panel.grid.minor = element_blank()) +
  facet_grid(cols = vars(PostDinucleotide))
```

### Figure S5D

Distribution of TadAWT-TadAEvo-ABE7.10 editing efficiency across the protospacer by post-dinucleotide context

```
abe_data %>%
  flushOutData(., "A") %>%
  filter(!is.na(PostDinucleotide)) %>%
  ggplot(aes(x = Position, y = Edit_norm, fill = PostDinucleotide)) +
  ylab("Percent Editing") +
  xlab("Position in Protospacer") +
  geom_bar(stat = "summary", fun.y = "mean", color  = "black", alpha = 0.7) +
  stat_smooth(aes(outfit=tmp_fit<<-..y..), span = 0.4, color = "black") + 
  geom_point() +
  scale_y_continuous(limits  = c(0, 1), breaks = seq(0,1, 0.2), labels = scales::percent_format()) +
  scale_x_continuous(breaks = seq(0, 20, 2)) +
  coord_cartesian(xlim =  c(1,20), clip = "on")  +
  scale_fill_manual(values = c("All" = "white", "AA" = "#4daf4a", "AC" = "#377eb8", "AG" = "grey", "AT" = "#e41a1c")) +
  labs(fill = "Post-dinucleotide") +
  theme_bw(base_size = 18) +
  theme(aspect.ratio = 1/2,
        panel.grid.major = element_blank(), panel.grid.minor = element_blank()) +
  facet_grid(cols = vars(PostDinucleotide))
```

### Figure S5E

Summary of linear model of rAPOBEC1-BE4 editing efficiency as a function of post-dinucleotide context

```
cbe_data %>%
  flushOutData(., "C")  %>%
  lm(Edit_norm ~ 0 + PostDinucleotide, data = .) %>%
  summary() %>%
  .[[4]] %>%
  as.data.frame() %>%
  mutate(PostDinucleotide = c("All", "CT", "CC", "CA", "CG")) %>%
  dplyr::rename(`Average Editing` = Estimate) %>%
  dplyr::select(PostDinucleotide, everything()) %>%
  mutate(`Average Editing` = scales::percent(`Average Editing`),
         `Std. Error` = scales::percent(`Std. Error`),
         `t value` = signif(`t value`, 3),
         `Pr(>|t|)` = signif(`Pr(>|t|)`, 3)
  )
```

```
##                     PostDinucleotide Average Editing Std. Error t value
## PostDinucleotideAll              All          17.39%       1.4%   12.00
## PostDinucleotideCT                CT          21.49%       2.7%    7.92
## PostDinucleotideCC                CC          19.60%       2.7%    7.22
## PostDinucleotideCG                CA           8.47%       4.4%    1.93
## PostDinucleotideCA                CG          16.95%       2.7%    6.24
##                     Pr(>|t|)
## PostDinucleotideAll 3.94e-30
## PostDinucleotideCT  1.12e-14
## PostDinucleotideCC  1.54e-12
## PostDinucleotideCG  5.34e-02
## PostDinucleotideCA  7.89e-10
```

### Figure S5F

Summary of linear model of TadAWT-TadAEvo-ABE7.10 editing efficiency as a function of post-dinucleotide context

```
abe_data %>%
  flushOutData(., "A")  %>%
  lm(Edit_norm ~ 0 + PostDinucleotide, data = .) %>%
  summary() %>%
  .[[4]] %>%
  as.data.frame() %>%
  mutate(PostDinucleotide = c("All", "AT", "AC", "AA", "AG")) %>%
  dplyr::rename(`Average Editing` = Estimate) %>%
  dplyr::select(PostDinucleotide, everything()) %>%
  mutate(`Average Editing` = scales::percent(`Average Editing`),
         `Std. Error` = scales::percent(`Std. Error`),
         `t value` = signif(`t value`, 3),
         `Pr(>|t|)` = signif(`Pr(>|t|)`, 3)
  )
```

```
##                     PostDinucleotide Average Editing Std. Error t value
## PostDinucleotideAll              All          12.56%      1.34%    9.35
## PostDinucleotideAT                AT          10.66%      3.24%    3.29
## PostDinucleotideAC                AC          20.14%      2.84%    7.10
## PostDinucleotideAG                AA           8.98%      2.35%    3.82
## PostDinucleotideAA                AG          13.48%      2.73%    4.94
##                     Pr(>|t|)
## PostDinucleotideAll 1.00e-19
## PostDinucleotideAT  1.04e-03
## PostDinucleotideAC  2.85e-12
## PostDinucleotideAG  1.46e-04
## PostDinucleotideAA  9.47e-07
```

## Figure S6

Comparison of context preferences from meta-analysis to BE-hive and the basis of the Honeycomb scoring algorithm

### Figure S6A

BE-Hive predicted editing efficiency for base edits in meta-analysis grouped by preceding dinucleotide context

```
ABE_guides_summarized %>%
  mutate(Edit_norm = Edit_norm) %>%
  ggplot(aes(x = Dinucleotide, y= pG)) +
  geom_boxplot() +
  scale_fill_gradientn(colors = colors) +
  theme_bw(base_size = 18) +
  geom_point(pch =21, size =3, alpha = 0.6) +
  labs(fill = "Position") +
  xlab("Dinucleotiode") +
  ylab("Predicted Editing Efficiency (BE-hive)") +
  theme(aspect.ratio = 0.5, panel.grid = element_blank())
```

### Figure S6B

BE-Hive predicted editing efficiency for base edits in meta-analysis grouped by preceding dinucleotide context

```
# Plot data
BE4_guides_summarized %>%
  mutate(Edit_norm = Edit_norm) %>%
  ggplot(aes(x = Dinucleotide, y= pT)) +
  geom_boxplot() +
  scale_fill_gradientn(colors = colors) +
  theme_bw(base_size = 18) +
  geom_point(pch =21, size =3, alpha = 0.6) +
  labs(fill = "Position") +
  xlab("Dinucleotiode") +
  ylab("Predicted Editing Efficiency (BE-hive)") +
  theme(aspect.ratio =  0.5, panel.grid = element_blank())
```

### Figure S6C

Logistic regression context motif weights of ABE7.10 and BE4 first demonstrated in Arbab & Shen et al.

```
context_weights %>%
  filter(enzyme == "ABE7.10") %>%
  mutate(`0` = c(0, 0, 0, 0)) %>%
  dplyr::select(base, `-3`:`-1`, `0`, `1`:`3`) %>%
  column_to_rownames("base") %>%
  as.matrix() %>%
  ggseqlogo(., method='custom', seq_type='dna') +
  scale_x_continuous(breaks = 1:7, labels = c("-3", "-2", "-1", "Target", "1", "2", "3")) +
  # scale_y_continuous(limits = c(-1, 1)) +
  theme_bw(base_size = 14) +
  geom_hline(yintercept = 0, linetype = "dashed") +
  ylab("BE-Hive logistic regression weights") +
  ggtitle("ABE7.10 Logistic regression weight by sequence context") +
  theme(panel.grid = element_blank())
```

### Figure S6D

Logistic regression context motif weights of ABE7.10 and BE4 first demonstrated in Arbab & Shen et al.

```
context_weights %>%
  filter(enzyme == "BE4") %>%
  mutate(`0` = c(0, 0, 0, 0)) %>%
  dplyr::select(base, `-3`:`-1`, `0`, `1`:`3`) %>%
  column_to_rownames("base") %>%
  as.matrix() %>%
  ggseqlogo(., method='custom', seq_type='dna') +
  scale_x_continuous(breaks = 1:7, labels = c("-3", "-2", "-1", "Target", "1", "2", "3")) +
  theme_bw(base_size = 14) +
  geom_hline(yintercept = 0, linetype = "dashed") +
  ylab("BE-Hive logistic regression weights") +
  ggtitle("BE4 Logistic regression weight by sequence context") +
  theme(panel.grid = element_blank())
```

### Figure S6E

Logistic regression weights for ABE7.10 and BE4 by position of target base in the protospacer

```
position_weights %>%
  filter(position >=1 & position <=20) %>%
  ggplot(aes(x = position, y = position_weight, fill = enzyme)) +
  geom_bar(stat = "identity", color = "black") +
  scale_x_discrete(labels = 1:20, breaks = 1:20, limits = 1:20) +
  theme_bw(base_size = 14) +
  scale_fill_brewer(palette = "Set1") +
  facet_grid(rows = vars(enzyme))+
  geom_hline(yintercept = 0, linetype = "dashed") +
  ylab("BE-Hive logistic regression weight") +
  xlab("Position in protospacer of target base") +
  labs(fill = "Enzyme") +
  ggtitle("Logistic regression weight by position in protospacer") +
  theme(panel.grid = element_blank())
```

### Figure S6F

Calculations of Honeycomb score

## Figure S7

Mapping of sgRNAs used in this work to the genomic loci of (a) B2M, (b) CD3D, (c) CD3E, (d) CD3G, (e) CD247, (f) TRAC.

## Figure S8 - S14

Mapping of sgRNAs used in this work to the crystal structure

## Figure S15

Disrupting the immunoinhibitory protein CISH with BE-splice guides in K562 cell line

### Figure S15A

Map of sgRNAs at CISH locus

### Figure S15B

Comparison of editing efficiencies across ABE7.10, ABE8e, and BE4

```
CISH %>%
  ggplot(aes(x = Enzyme, y = Edit, fill = Enzyme)) +
  geom_bar(stat = "summary", fun.y = "mean", position = "dodge", color = "black") +
  scale_fill_brewer(palette = 1) +
  geom_point(position = position_dodge2(0.2), color = "black", size = 3) +
  # facet_grid(cols = vars(Enzyme), space = "fixed", scale = "free") + 
  scale_y_continuous(limits = c(0,1.3), breaks = seq(0,1,0.2), labels = scales::percent_format()) +
  ylab("Editing Efficiency") +
  xlab("")+
  theme_bw(base_size = 28) +
  theme(axis.text.x = element_text(hjust = 1, angle = 45),
        legend.position = "none",
        panel.grid = element_blank(),
        aspect.ratio = 2)
```

```
CISH %>%
  lm(Edit ~ Enzyme, data = .) %>%
  aov() %>%
  TukeyHSD() %>%
  .$Enzyme
```

```
##                   diff        lwr        upr        p adj
## ABE8e-ABE7.10  0.81625  0.5480082 1.08449181 4.688143e-07
## coBE4-ABE7.10  0.57750  0.3092582 0.84574181 6.318474e-05
## coBE4-ABE8e   -0.23875 -0.5069918 0.02949181 8.688619e-02
```

### Figure S15C

Relative splice site expression of all exon spanning Taqman assays across different treatments

```
taqman4 %>%
  filter(Junction != "β-Actin") %>%
  #filter(Junction == "Ex.1a – Ex.2" | Junction == "Ex.2 – Ex.3") %>%
  ggplot(aes(x = Guide, y = `2^(-ΔΔCt)`, fill = Junction)) +
  geom_bar(stat = "summary", fun.y = "mean", position = "dodge", color = "black") +
  scale_fill_brewer(palette = "Dark1") +
  # geom_errorbar( position = position_dodge(), colour="black") +
  geom_point(position = position_dodge(0.9), color = "black", size = 3) +
  geom_hline(yintercept = 1, linetype = "dashed") +
  facet_grid(cols = vars(Enzyme), space = "fixed", scale = "free") + 
  xlab("")+
  ylab("Relative Splice Expression") +
  theme_bw(base_size = 28) +
  theme(axis.text.x = element_text(hjust = 1, angle = 45),
        panel.grid = element_blank(),
        aspect.ratio = 0.9)
```

### Figure S15D

Expression of splice site junctions shown as the fold expression of β-Actin

```
taqman4 %>%
  filter(Junction != "β-Actin") %>%
  #filter(Junction == "Ex.1a – Ex.2" | Junction == "Ex.2 – Ex.3") %>%
  ggplot(aes(x = Guide, y = `Fold of B-Actin`, fill = Junction)) +
  geom_bar(stat = "summary", fun.y = "mean", position = "dodge", color = "black") +
  scale_fill_brewer(palette = "Dark1") +
  # geom_errorbar( position = position_dodge(), colour="black") +
  geom_point(position = position_dodge(0.9), color = "black", size = 3) +
  facet_grid(cols = vars(Enzyme), space = "fixed", scale = "free") + 
  xlab("")+
  ylab("Fold expression of β-Actin") +
  theme_bw(base_size = 28) +
  theme(axis.text.x = element_text(hjust = 1, angle = 45),
        panel.grid = element_blank(),
        aspect.ratio = 0.9)
```

### Figure S15E

Uncropped image of gel presented in figure 6E

## Figure S16

### Figure S16A

Map of CD3E gRNAs and Taqman assay probes

### Figure S16B

Taqman assays of exon 1-2 and exon 6-7 junctions

```
experimental_data %>%
  filter(!is.na(`CD3E_Exon_1-2`)) %>%
  gather(Junction, value, `CD3E_Exon_1-2`:`CD3E_Exon_6-7`) %>%
  mutate(Junction = gsub("_", " ", gsub("CD3E_", "", Junction))) %>%
  ggplot(aes(x = Guide_Name, y = value, fill = Junction)) +
  geom_bar(stat = "identity", position = "dodge", color = "black") +
  scale_fill_brewer(palette = 1) +
  geom_point(position = position_dodge(1), size = 3) +
  scale_y_continuous(limits = c(0, 1.2), breaks = seq(0,1,0.2), labels = scales::percent_format()) +
  ylab("Percent Expression of Negative Control") +
  xlab("") +
  geom_hline(yintercept = 1, linetype = "dashed", color = "black") +
  theme_bw(base_size = 18) +
  theme(panel.grid = element_blank(),
        axis.text.x = element_text(hjust = 1, angle = 45))
```

### Figure S16C

Remaining percentage of WT allele and protein expression

```
experimental_data %>%
  filter(!is.na(`CD3E_Exon_1-2`)) %>%
  dplyr::mutate(`WT Allele` = `C`/100,
                `Protein Expression` = Flow) %>%
  gather(Metric, value, c(`WT Allele`,`Protein Expression`)) %>%
  mutate(Metric = factor(Metric, levels = c("WT Allele","Protein Expression"))) %>%
  ggplot(aes(x = Guide_Name, y = value, fill = Metric)) +
  geom_bar(stat = "identity", position = "dodge", color = "black") +
  scale_fill_brewer(palette = 2) +
  geom_point(position = position_dodge(1), size = 3) +
  scale_y_continuous(limits = c(0, 1.2), breaks = seq(0,1,0.2), labels = scales::percent_format()) +
  ylab("Percent Expression of Negative Control") +
  xlab("") +
  labs(fill = "") +
  geom_hline(yintercept = 1, linetype = "dashed", color = "black") +
  theme_bw(base_size = 18) +
  theme(panel.grid = element_blank(),
        axis.text.x = element_text(hjust = 1, angle = 45))
```

## Figure S17

### Figure S17A

Quantification of the relative expression of each band normalized to β-actin and the AAVS1 contro

```
CISH %>%
  gather(Isoform, Expression, `Normalized_CISH-1`:`Normalized_CISH-2`) %>%
  ggplot(aes(x = Guide, y = Expression, fill = Isoform)) +
  geom_bar(stat = "summary", fun.y = "mean", position = "dodge", color = "black") +
  scale_fill_brewer(palette = 3) +
  # geom_errorbar( position = position_dodge(), colour="black") +
  geom_point(position = position_dodge(0.9), color = "black", size = 3) +
  facet_grid(cols = vars(Enzyme), space = "fixed", scale = "free") + 
  scale_y_continuous(breaks = seq(0,1.4,0.2), labels = scales::percent_format()) +
  geom_hline(yintercept = 1, linetype = "dashed") +
  ylab("Relative Protein Expression") +
  xlab("")+
  theme_bw(base_size = 28) +
  theme(axis.text.x = element_text(hjust = 1, angle = 45),
        panel.grid = element_blank(),
        aspect.ratio = 0.9)
```

### Figure S17B

Scatter plot of normalized protein expression vs. the remaining WT allele after treatment, showing a strong correlation between editing and protein knockout

```
label = CISH %>%
  filter(!grepl("AAVS1", Guide)) %$%
  cor.test(x = 1-.$Edit, y = .$`Normalized_CISH-1`) %$%
  {paste0("r = ", signif(.$estimate, 3),", P = ", signif(.$p.value, 3))}

CISH %>%
  filter(!grepl("AAVS1", Guide)) %>%
  ggplot(aes(x = 1-Edit, y = `Normalized_CISH-1`)) +
  geom_smooth(method = "lm") +
  geom_point() +
  theme_bw(base_size = 18) +
  scale_y_continuous(limits = c(0,1.4), breaks = seq(0,1.4,0.2), labels = scales::percent_format())+
  scale_x_continuous(limits = c(0,1), breaks = seq(0,1,0.2), labels = scales::percent_format())+
  annotate(geom = "label", label = label, x = 0.25, y = 1.1) +
  ylab("Normalized Protein Expression") +
  xlab("Percent of WT base remaining") +
  theme_bw(base_size = 18) +
  theme(aspect.ratio = 1, panel.grid = element_blank())
```

### Figure S17C

Uncropped digital western blot for CISH.

### Figure S17D

Uncropped digital western blot for β-actin

### Figure S17E

Pilot experiment in T cells before the development of ABE8e with CISH targeting guide

## Figure S18

A standardized workflow for designing and testing base editor sgRNAs for gene disruption
